# Understanding how Eastern European migrants use and experience UK health services: a systematic scoping review

**DOI:** 10.1186/s12913-020-4987-z

**Published:** 2020-03-06

**Authors:** Viet-Hai Phung, Zahid Asghar, Milika Matiti, A. Niroshan Siriwardena

**Affiliations:** 1 Community and Health Research Unit (CaHRU), School of Health and Social Care, University of Lincoln, Lincoln, LN6 7TS UK; 2 School of Health and Social Care, University of Lincoln, Lincoln, LN6 7TS UK; 3 East Midlands Ambulance Service (EMAS) NHS Trust, Cross O’Cliff Court, Lincoln, LN4 2HL UK

**Keywords:** Eastern Europe, Migrant, Healthcare, Scoping review

## Abstract

**Background:**

The UK has experienced significant immigration from Eastern Europe following European Union (EU) expansion in 2004, which raises the importance of equity and equality for the recent immigrants. Previous research on ethnic health inequalities focused on established minority ethnic groups, whereas Eastern European migrants are a growing, but relatively under-researched group. We aimed to conduct a systematic scoping review of published literature on Eastern European migrants’ use and experiences of UK health services.

**Methods:**

An initial search of nine databases produced 5997 relevant publications. Removing duplicates reduced the figure to 2198. Title and abstract screening left 73 publications. Full-text screening narrowed this down further to 10 articles, with three more from these publications to leave 13 included publications. We assessed publications for quality, extracted data and undertook a narrative synthesis.

**Results:**

The included publications most commonly studied sexual health and family planning services. For Eastern European migrants in the UK, the most commonly cited barriers to accessing and using healthcare were limited understanding of how the system worked and language difficulties. It was also common for migrants to return to their home country to a healthcare system they were familiar with, free from language barriers. Familial and social networks were valuable for patients with a limited command of English in the absence of suitable and available interpreting and translating services.

**Conclusions:**

To address limited understanding of the healthcare system and the English language, the NHS could produce information in all the Eastern European languages about how it operates. Adding nationality to the Electronic Patient Report Form (EPRF) may reveal the demand for interpretation and translation services. Eastern European migrants need to be encouraged to register with GPs to reduce A&E attendance for primary care conditions. Many of the issues raised will be relevant to other European countries since the long-term outcomes from Brexit are likely to influence the level of Eastern European and non-Eastern European migration across the continent, not just the UK.

## Background

While equity is one of the key principles of healthcare [1], research evidence suggests that it remains an unfulfilled aspiration for many migrant and minority ethnic groups around the world.

A North American systematic review found that community navigators can simplify the process of using the Canadian healthcare system. Community navigators also facilitated the settlement, adaptation, and integration of immigrant and refugee women from over 80 countries into Canadian society [2].

A German study found that people from a migrant background were less likely to have a GP, one explanation being those with a migrant background had trouble understanding the German healthcare system, which was significantly different to what they had experienced before arriving in Germany. Not having a GP may increase the likelihood of using healthcare services inappropriately [3].

In a Swedish study, healthcare staff felt that there were complex challenges in providing patient care for migrants: diversity, language barriers, problems navigating the Swedish healthcare system and the need to use interpreters during patient encounters. Most patients came from the Middle East and North Africa (47%) and Sub-Saharan Africa (24%). To overcome these problems, number of key recommendations were proposed: translating key documents into migrants’ native languages; increased staff cultural competency training; and community-based educational outreach programmes to improve health literacy [4].

There also appears to be a disconnect between minority ethnic and migrant groups and the UK National Health Service (NHS). A scoping review identified differences in access to, and experience of, diabetes information and services by British Bangladeshis. Patients had limited knowledge of how to manage diabetes and were frequently using friends and family as informal interpreters [5]. A study of antenatal care among Black and Minority Ethnic (BME) women found that a lack of familiarity with the healthcare system meant that the women did not know where to go for information; limited language skills necessitated the use of professional interpreters, which were often not provided leading to an inappropriate reliance on family members, including children, to interpret; women perceived that healthcare staff did not understand the negative impact of language and communication problems [6]. Similar findings arose from a systematic review of minority ethnic groups and access to diabetes services in the UK. The predominant groups in this systematic review were: Black Caribbean; Bangladeshi; Black African; Pakistani; and Muslim Kashmiri [7].

Of increasing importance to the UK is the impact of more recent Eastern European migration on the NHS. The A8 former Communist countries (Czech Republic, Estonia, Hungary, Latvia, Lithuania, Poland, Slovakia and Slovenia), as well as Malta and Cyprus joined the European Union (EU) in 200; the A2 countries (Romania and Bulgaria) joined in 2007 (with transitional arrangements in the UK until 2013), followed by Croatia in 2013. Citizens from these countries have been taking up new rights to freedom of movement to live and work anywhere in the EU.

In 2017, the UK had almost two million residents from the A8 and A2 countries [8], which has resulted in legislative and policy responses. The Equality Act 2010 [9] requires public and private services to demonstrate equal treatment across all areas of employment and delivery of care; including access to care provision. The Equality Delivery System (EDS1 and EDS2) gives practical advice to the UK’s National Health Service (NHS) organisations on how to comply with The Equality Act 2010 [10, 11].

The is increasing research evidence about how these more recent Eastern European migrants experience the UK NHS. A study in Warrington in North West England found that while GP registration was high, it was also the case that Eastern Europeans found it difficult to navigate the NHS. They were dissatisfied with GPs, who were perceived to not take their health concerns, or those of their children, seriously enough [12]. Polish and Roma in Barking and Dagenham on the London/Essex border had low levels of awareness of health and language services. Language barriers, allied to the lack of understanding of the workings of the NHS and lack of staff cultural competency, hindered their access to healthcare services [13].

This systematic scoping review aimed to build on existing knowledge of how Eastern European migrants use and experience UK healthcare services to inform service delivery improvements this population.

## Methods

### Design

A systematic scoping review was used to explore the broad research question of what evidence there was of how Eastern European migrants use and experience UK health services, and generally followed the Arksey and O’Malley framework five-point framework: identifying a research question; identifying relevant studies; selecting studies; charting data; and collating, summarising and reporting results [14]. This model has been endorsed by other researchers [15, 16].

### Search terms

Search terms were selected to be as broad as possible covering three domains: health; geography and migration. Within the health domain, search terms encompassed different aspects of healthcare including emergency, primary and hospital care, as well as health generically. The geographic domain included Europe and Eastern Europe. The migration domain included references to migrants and immigrants. Publications with European migrants, as well as publications located in Europe and Eastern Europe, were filtered out in the final stage of screening.

### Databases

Searches took place from November 2016 to February 2017 supported by the University of Lincoln health subject librarian [17], with nine databases selected to cover as wide a range as possible: Academic Search Complete; Cumulative Index to Nursing and Allied Health Literature (CINAHL); MEDLINE; PsycInfo; International Bibliography of the Social Sciences (IBSS); Scopus; Web of Science; Health Business Elite (HBE); and Health Management Information Consortium (HMIC).

### Inclusion and exclusion criteria

Inclusion criteria included: peer reviewed; English language; published from 1980 to 2016. This timeframe was chosen in order to capture all the significant geopolitical changes within Europe, including the fall of Communism. There were no restrictions on study type or types of interventions because we wanted as broad a coverage as possible [14]. We excluded foreign language publications because of the cost and time involved in translation. Grey literature was also excluded. We applied as closely as possible, given the different formats, the same search terms and combinations (Table S1) to the nine databases. The results represent publications identified with all the migrant, health and geographical terms combined. The output from these combined searches were exported to Endnote version 8.

### Title and abstract screening

After removing duplicates, publications were subject to a two-stage screening process. Firstly, publications were screened by title and abstract [18] using a scoring system, in which studies were included if title or abstract met all of the following criteria each scored as “1” or “0”: main focus on health; focus only on Europe; and including only European migrants. Publications scoring “3” went to full-text review.

Because there were no restrictions on study type or type of intervention, as suggested by Arksey and O’Malley [14] we had to find an alternative way to include and exclude publications. To that end, we agreed on a scoring system. Studies were included if their title or abstract met all of the following criteria: main focus was on health; focus only on Europe; and included only regular European migrants. Each publication scored “1” or “0” and given “1” for each if the main focus was on health, solely on Europe or Eastern Europe, or if they included only regular European or Eastern European migrants. To go to full-text screening, each publication had to score “3”. Two of these contained sufficiently useful information to be kept as background material, despite only scoring “2”.

### Full-text screening

Publications included after full-text screening contained at least three of the following terms: “health”; “emergency or emergencies”; “hospital”; “ambulance”; and “primary care”. Each publication scored a “1” for each inclusion criterion met. They also had to focus on both Europe and on European or Eastern European migrants. To go forward to the final stage, each publication had to score at least “3” out of the five health terms and “2” on the European or Eastern European terms. These stricter criteria were designed to effectively screen the remaining full-text articles for relevance.

The “health” term by its very nature was wide and terms relating to all sectors of healthcare were included. A consequence of using “European or Eastern European” was that some publications focused on migrants in continental Europe, while others related to non-Eastern European migrants in the UK. These studies were excluded. Publications that were not research papers were also excluded at this stage.

### Quality assessment

The publications remaining after the two-stage screening process were assessed for risk of bias. Different quality assessment tools (checklists) were used for qualitative [19], quantitative [20], and mixed methods studies [21].

The seven quantitative studies were all cross sectional so we used the Joanna Briggs Instititute (JBI) eight-point checklist for cross-sectional studies [20] covering: inclusion criteria; valid measurement of exposures; objective measurement of the condition; and whether confounding factors were identified.

To the five qualitative studies we applied the ten-point CASP [19] checklist which included: appropriateness of qualitative methods; recruitment; data collection; and value that the study added. Within each of these ten points are a number of hints or sub-themes which were also examined.

The Mixed Methods Appraisal Tool (MMAT) from McGill University [21] with its five sections checklist was applied to the one mixed methods study. The first compulsory section of the checklist focuses solely on the qualitative element. The next three, only one of which is mandatory, relate to the quantitative elements. The final section assesses the extent to which the mixed methods approach addresses the research question.

## Results

Applying these search terms across the nine databases produced 5997 results (Table S1).

**Fig. 1:**
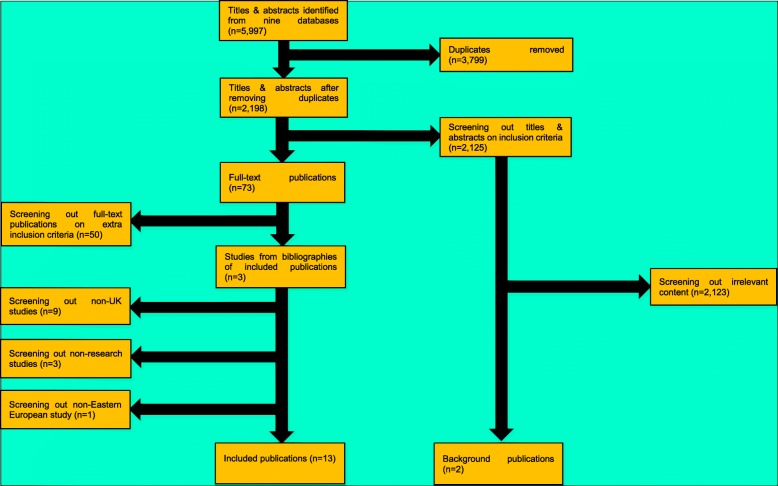
Flow chart of the data extraction process

After removing duplicates, there were 2195 publications from the original 5997. Title and abstract screening excluded 2122 publications to leave 73 publications needing full-text screening. Of the 2122 publications that were excluded in the title and abstract screening, two were kept as background, despite not meeting all the inclusion criteria. The subsequent full-text screening filtered out a further 50 publications to leave 23 articles. An editorial was excluded because it was not a research paper but this revealed three more studies [22–24] from a programme of work [25] on sexual risk-taking behaviour among Eastern European men that had already been included. Two other articles were also excluded as not being research papers. This brought the total of included publications to 23. We excluded nine of these 23 studies because they did not focus on migrants to the UK. One study was excluded because it was about Italian and not Eastern European migrants in the UK. There remained 13 publications (Fig. 1), all focusing on Eastern European migrants’ use of healthcare in the UK to be analysed further.

### Charting

We charted the following information: author and year; aims & objectives; population; methods; key results; discussion; and limitations (Table S2). Four of these were from the programme of work relating to risk-taking sexual behaviour among Eastern European men [22–25]. Of the remaining nine publications, five focused on maternal health [26–30], two related to primary care [31, 32], one was a study on the health of Eastern European children [33], while one study examined ED use among Polish migrant workers [34]. We did not focus on the type of study or intervention so this information was not charted.

### Quality assessment

There were five qualitative studies [27–30, 33]. Only one of the five studies [30] addressed all 10 points on the checklist (Table S3).

All the quantitative studies included were cross-sectional [22–26, 31]. The Bray study, because it was a single case study, did not adjust for confounding factors [26]. Four studies met all of the criteria on the JBI checklist [22–25] but some did not meet some key conditions (Table S4). One study did not focus on any specific condition so did not meet the checklist requirement for measuring the condition [31].

There was one mixed methods study in the final 13 publications [32]. The study addressed all checklist points relevant to it but did not include a randomised control trial, which is why this section was left blank (Table S5).

### Synthesis

After reading the 13 remaining publications in full, we used narrative synthesis to organise findings into the following inter-connected themes: access and use of healthcare services; expectations and understanding of the healthcare system; returning to their homeland for healthcare; language barriers and communication problems; and social networks.

### Access and use of healthcare services

The most commonly cited, often linked, barriers to accessing and using healthcare for Eastern European migrants in the UK were limited understanding of the way the NHS worked [28, 29, 31, 34] and language barriers [28, 29, 31, 33, 34]. Limited language skills, alongside limited provision of information in their native language, further complicated the process of accessing and using healthcare [30, 33]. Sometimes, where Eastern European women needed an interpreter, services were not available, which led staff to rely on family and/or friends, or even children to translate [26].

Published studies on healthcare service use, most commonly related to sexual health [22–25], family planning [26–28, 30] and child health services [29, 33]. One of the studies found that, overall, men were more likely than women to have at least one acute STI diagnosed at their clinic visit(s): 27.3% (95% CI 26.5% to 28.0%) versus 15.6% (95% CI 15.1% to 16.2%), respectively (*p* < 0.001) [25].

Another study found that while the incidence of prior sexually transmitted infections (STIs) are lower than in the general British population, Eastern European migrants, especially males, report high rates of behaviours associated with increased risk of HIV and STI transmission. These included: recreational drug use (aOR 1.37, 95% CI 1.01 to 1.87), drinking alcohol on average three or more days a week (aOR 1.62, 95% CI 1.14 to 2.28) and anal sex (aOR 1.89, 95% CI 1.35 to 2.64). The reported HIV prevalence of 1.1% is much higher than the estimated prevalence of 0.09% in the general British population [24].

Another study examined why Polish people visited GPs. Women were, on average, significantly more likely to visit their GP than men, especially among 25–34 (2.46 visits to 1.89) and 35–44 year-olds (3.11 visits to 2.35 visits) (*p* = 0.0038). Overall, the most common conditions that patients presented with were respiratory problems (33% of visits), especially upper respiratory tract infections (24% of visits). Musculoskeletal pain was also common overall (12.6% of visits), especially among 25–34 and 35–44 year-olds. The reasons for visiting the GP were not significantly associated with age [31].

### Expectations and understanding of the healthcare system

The healthcare expectations of Eastern European migrants in the UK were influenced by what they had been used to in their country of origin. For instance, waiting times were longer in the UK than they had been used to [27, 28, 30, 33]. Eastern European migrant women were not used to health visitors [29], while screening procedures during pregnancy were often markedly different [30]. Polish women in London complained of long waiting times and poor quality local healthcare [27, 28, 30, 32], which prompted them to return home for treatment [28, 30]. Some Polish women welcomed the healthcare choices available to them, while others were bewildered by them [27, 29]. The mismatch between expectations and reality was not always negative. For instance, some Eastern European migrant women in the UK were pleasantly surprised that screening was simpler than they had been used to in their country of origin and that blood tests were free [30].

Linked to the mismatch in expectations was the limited understanding of how the UK healthcare system worked, particularly what healthcare services they were entitled to and when they were meant to use them [29–31, 33, 34]. Some used emergency services for non-emergency conditions [29, 34]. One UK study found that recent Polish migrants were more likely to use A&E inappropriately compared to the indigenous population. The study found that ED attendances at a hospital in Telford, a town in the UK West Midlands, increased from an average of 134 from 2000 to 2003 to 357 in 2005. Of these 357, 152 (43%) were not registered with a GP. The overall rate of ED attendance for unregistered patients was 7.4% [34].

### Returning home for healthcare

Among the 13 final publications, a common theme was that Eastern European migrants often returned from the UK to their countries of origin to use healthcare services [26–28, 30, 32, 33]. There were push and pull factors that drove the decision to return to their homeland to seek treatment. The push factors drawing them away from seeking treatment in the UK included: longer waiting times in the UK [28, 33]; limited access to specialists [33], not being registered with a GP [30] and limited English language skills, which complicated their interactions with UK healthcare services [28, 30]. Negative opinions about UK healthcare provision, compared to what they had been used to before, also underpinned why some Eastern European migrants preferred to return home [27, 28, 30, 32], sometimes to get a second opinion on the treatment they had already received in the UK [30].

Many Eastern European migrants were pulled back home to seek medical treatment because of familiarity with, and trust in, the healthcare system there [28, 30, 33], as well as retaining their social networks (both personal and healthcare related) [28, 32, 33]. As longer waiting times were viewed negatively, so the perceived shorter waiting times back in their homeland were a significant pull factor [28]. Moreover, the language barriers that constrained their experiences in using healthcare in the UK would be absent if they returned back to their homeland [32].

### Language and communication barriers

Language and communication barriers, to some extent connected with the four other main themes identified in this review. These influenced the extent to which Eastern European migrants accessed healthcare services [28–30, 32, 34] and their (sometimes negative) experiences of it [26, 30]. Language had a more direct influence on their understanding of the UK healthcare system as Eastern European migrants in the UK sometimes needed familial and social networks, including children to translate written material [33] and mediate in healthcare encounters before interpreters became available or in the absence of suitable interpreters [26, 28]. One study found that some Eastern European migrant women felt that doctors in the UK stigmatised them for their limited command of English [30]. This tied into concerns about the availability and suitability of interpreting services compounding the language barrier [26, 30].

### The role of social networks

Familial and social networks, including children, played a crucial role in Eastern European migrants’ use of healthcare in the UK, these networks helping Eastern Europeans better understand what services were available and appropriate to use [32, 33]. Conversely, limited networks restricted access to information and hampered their ability to use appropriate services for their needs [33]. Social networks were also an option when the appropriate interpretation or translation services were unavailable to Eastern European migrants with a limited command of English during encounters with healthcare staff [26, 28].

## Discussion

### Key findings

Eastern European migrants often returned to their country of origin to use healthcare services. In doing so, they used their lay referral network [35] to access healthcare services in their home country and language, often combining this with social visits to family and friends. The availability of low cost flights and the understandable preference for familiar surroundings and networks facilitated the decision to return home [28, 33]. The decision also reflected dissatisfaction with UK healthcare in general or specific aspects of it, for example, waiting times.

Limited [29, 30, 33] or misleading [29] information about entitlements to services aggravated the problem of a mismatch between healthcare expectations and the reality of healthcare experiences for Eastern European migrants in the UK. It also reflected a lack of familiarity with the UK healthcare system, which was sometimes compounded by language barriers. Moreover, the language difficulties sometimes led to a perceived stigmatisation from doctors in their healthcare encounters, especially in maternal health [26]. Variable quality and provision of interpretation and translation services also prompted a reliance on family or social networks during encounters with healthcare staff [26, 33, 36].

Language barriers interacted with the other themes and was another factor explaining why some Eastern Europeans did not access the healthcare services they needed or had unsatisfactory healthcare experiences. A limited command of English may have affected understanding of how the NHS works [37]. This may be aggravated by a limited availability of information about the NHS in their native language. Eastern Europeans with a limited command of English need interpreters, but if no interpreters are available, they rely on relatives and/or friends to interpret [26, 33, 36].

There was an association between low GP registration and erroneous ED use [34]. Eastern European migrants may not realise the importance of the GP within the UK NHS, and without a GP, they may have little option but to access healthcare through the ED [33, 34]. Migrants attending ED with ‘symptom trivia’ [38] could have been treated in primary care if they were registered with a GP. High ED attendance rates by migrants could be due to their limited understanding of how the UK NHS works and linked to the recency of their migration to the UK. However, Given the negative opinions of GPs expressed by some Eastern European migrants [12, 26], access and experience will also need to improve before ED use is reduced.

### Implications for future research, policy and practice

The systematic approach can be replicated, and used for other population groups and geographical areas. Undertaking such a systematic scoping review can help to inform policy, practice and further research by establishing a credible, quality assessed evidence base [39]. Healthcare providers need to understand the push and pull factors that prompt Eastern European migrants in the UK to return home to use healthcare. By understanding these reasons, UK service providers could provide practical measures to improve access to and experience of healthcare. One way of doing so would be outreach using culturally appropriate material to inform particular groups about the services available, and in particular those with language barriers or unfamiliar with how the UK healthcare system operates.

By doing so, service providers may be able to better manage healthcare expectations of Eastern European migrants in the UK and enable them to understand the relative costs and benefits of accessing treatment in the UK compared with their home country. Staff cultural competency training may reduce the likelihood of stigmatising behaviour towards patients, which can improve the healthcare experience of Eastern European migrants. This, in turn, could reduce the likelihood that Eastern European migrants return home for treatment.

To narrow the disparity in waiting times between the UK and some Eastern European countries to make seeking treatment in their country of residence more attractive would require greater resources for the NHS. Despite such efforts, Eastern European migrants may still choose to return home to use healthcare services because it enables visits to family and friends in their home country.

The use of familial and social networks to help Eastern European migrants use healthcare services in the UK poses dilemmas for service providers. Family and friends may be used to translate written material and provide reassurance in encounters with healthcare professionals. This may be vital for those with a limited command of English and a limited understanding of how the UK healthcare system works but the use of familial and social networks to translate during interactions with healthcare staff may increase the possibility that translations are biased, inaccurate, incomplete or all three.

There is a dearth of information published in Eastern European languages to help patients make use of the healthcare in the UK. Providing more information about appropriate healthcare use in different languages could be cost-effective if they led to more appropriate use of services. To achieve this, the NHS has to find more effective ways of encouraging Eastern European migrants to register with GPs so that the Emergency Department is a last, rather than the only, option to access healthcare. Part of the problem lies here in the transient nature of some Eastern European migrants, which reduces the incentive to register with GPs [30].

The availability and quality of professional interpreting services is unsatisfactory and provision of high quality translation and interpretation services could facilitate better patient-provider communication and help Eastern European migrants make more informed healthcare decisions. Better provision of interpretation and translation services have to be underpinned by greater consistency and integration in the collection and application of ethnicity monitoring data [40].

Eastern European migrants are classified as White Other in the current form of the Electronic Patient Report Form (EPRF). This classification does not capture the diversity of this heterogeneous group. Adding nationality may help to overcome this and may highlight the extent of need for professional intepretation and translation services. It may also be helpful if databases from different sectors of the NHS could be linked. This could simplify the process of diagnosis and treatment for healthcare professionals by enabling them to see past medical history, which could provide useful context. Registering with GPs could facilitate effective information sharing between the relevant agencies.

In terms of distributing the information about how the NHS works, it may be worth targeting community centres for particular nationalities or churches, which play an important part in the lives of some Eastern European communities. In some towns and cities, it is also common for some factories to have a high concentration of mainly Eastern European agency workers. It may also be worth targeting native language TV channels, which Eastern European migrants may feel more comfortable with while they are learning the language. Healthcare organisations may also wish to develop a dialogue with Eastern European migrant communities, both through face-to-face meetings and through social media. Many community groups have Facebook and Twitter pages.

Such engagement may also enable them to make informed decisions about whether to use healthcare in their country of residence or origin. In relation to the latter, Eastern European migrants may also be attracted by low-cost travel, familiarity with the healthcare system, the absence of a language barrier and the prospect of visiting friends and family. Economic prosperity, greater employment opportunities and higher wages may attract Eastern European migrants and that is something that other EU countries may have to anticipate.

While there is much that policy-makers and service providers can do to make healthcare services more accessible to Eastern European patients, some responsibility also has to be borne by the latter. The most basic would be to learn to speak the language of the country of residence sufficiently well to communicate with healthcare providers and understand written material. If migrants (from Eastern Europe and beyond) are learning to speak the language of their country of residence, then information about the healthcare system could be distributed in classes in their native language.

It is important to appreciate that migrants are not a homogeneous group. For example, many Eastern European migrants are younger working age adults, often more healthy and less likely to use the NHS. Some may have children, for whom they may need, for example, health visitors, paediatricians, etc. Other migrant groups may have an older profile, which means they may be more likely to fall ill and use the NHS more. There may also be issues around language and health literacy, which healthcare professionals may have to respond to. For instance, a systematic review found that to overcome issues of limited language and health literacy, healthcare professionals used audio-visual aids to improve comprehension, information retention, patient compliance and understanding if ethnic minority participants could not read or write in their first language [7].

There are also different levels of migration status that also need to be considered. Groups like asylum seekers are often hidden because they are not allowed to work until they receive refugee status. They may have complex health needs, as well as limited language skills and low understanding of how the healthcare system works. This is common to other migrant groups in the UK, but asylum seekers are harder to reach because they are not officially recognised by the Government. Other countries may need to examine how their asylum and immigration policies can keep these groups close to mainstream healthcare services.

All these issues are relevant not just to the UK, but to other EU countries too. The uncertain post-Brexit environment [41–44] may dissuade some Eastern Europeans from settling in the UK, with some preferring instead to go to mainland Europe. Uncertainty over whether they acquire the more secure settled status as opposed to the less secure pre-settled status [45] may prompt some Eastern Europeans in the UK to return home [46]. This may result in less demand in the UK for Eastern European interpreters and less need for material to be translated into Eastern European languages. However, the opposite may occur in other EU countries which may prove to be more attractive to Eastern European migrants than the UK after Brexit. Healthcare systems in EU countries may have to respond to increased migration of Eastern European migrants by providing more and better quality translation services. Moreover, to accommodate a significant influx of Eastern European migrants, more information about the healthcare system may need to be published in a number of Eastern European languages, both in written and electronic form.

Each country needs to respond to the continually changing demands on its healthcare system resulting from dynamic migration flows. While healthcare providers, given limited resources, cannot respond to each and every population group, effectively prioritising scarce resources is important.

### Strengths and limitations

We used a systematic approach, which can be applied to different healthcare settings, target population groups and geographical locations. We do not make any claims about representativeness of the studies in terms of the healthcare issues that are most salient for Eastern European migrants to the UK. Quality assessment minimised the risk of bias and suggested that included publications were methodologically robust. While the search encompassed a broad range of databases, it excluded grey literature and foreign language publications which may provide further valuable information. This review provides a snapshot of evidence at a particular moment in time which may change as more research is publsihed. The usefulness of the systematic scoping review to service providers and policy-makers depends to a large extent on what the long-term outcomes will be from Brexit. These long-term outcomes, unclear as they may be right now, may influence the nature and extent of the Eastern European population in the UK and other European countries.

## Conclusion

This systematic scoping review synthesised the literature on healthcare use and experiences of Eastern European migrants in the UK and enhances our understanding of the research evidence in this area. The method used can be adapted for different population groups, geographical locations and healthcare settings. Much of the research evidence around ethnic health inequalities in the UK focuses on previously established minority ethnic groups rather than more recent EU migrants. Brexit may of course affect Eastern European and non-Eastern European populations in the UK.

The findings have implications for NHS service providers, helping to ensure that Eastern European migrants are getting the best possible service. To achieve this NHS service providers need to engage with these communities to inform them of what services they are entitled to, how services are delivered, how they can access and use them appropriately. Communication should use a variety of methods including social media, traditional media, and focusing on places and times where they meet.

## Supplementary information

**Additional file 1: Table S1.** All nine databases with search terms and results.

**Additional file 2: Table S2.** Included publications.

**Additional file 3: Table S3.** Critical Appraisal Skills Programme (CASP) assessment of the included qualitative studies.

**Additional file 4: Table S4.** Joanna Briggs Institute (JBI) checklist for analytical cross-sectional studies.

**Additional file 5: Table S5.** Mixed Methods Appraisal Tool (MMAT).

## Data Availability

Not applicable.

## Abbreviations

A2: Accession countries that joined the EU in 2007 with transitional arrangements (Romania and Bulgaria)
A8: Eastern European Accession countries that joined the EU in 2004 (Czech Republic, Estonia, Hungary, Latvia, Lithuania, Poland, Slovakia, Slovenia)
ACS: Academic Search Complete
CASP: Critical Appraisal Skills Programme
CINAHL: Cumulative Index to Nursing and Allied Health Literature
EDS: Equality Delivery System
EPRF: Electronic Patient Report Form
EU: European Union
GUM: Genito-Urinary Medicine
HBE: Health Business Elite
HIV: Human Immunodeficiency Virus
HMIC: Health Management Information Consortium
IBSS: International Bibliography of the Social Sciences
JBI: Joanna Briggs Institute, Australia
MMAT: Mixed Methods Appraisal Tool
NHS: National Health Service
STI: Sexually Transmitted Infection
UK: United Kingdom
