# Supplementary material for: Understanding how Eastern European migrants use and experience UK health services: a systematic scoping review
Source: BMC Health Serv Res. 2020 Mar 6;20:173. doi: 10.1186/s12913-020-4987-z (PMC7059702; doi:10.1186/s12913-020-4987-z)
Supplement: Supplementary file 1 — Additional file 1: Table S1. All nine databases with search terms and results. [file 12913_2020_4987_MOESM1_ESM.docx]

**Table S1 All nine databases with search terms and results**

| **Search terms** | **Databases** | | | | | | | | | **Total** |
| --- | --- | --- | --- | --- | --- | --- | --- | --- | --- | --- |
|  | **ACS** | **Cinahl** | **Medline** | **PsycInfo** | **IBSS** | **Scopus** | **Web of Science** | **HBE** | **HMIC** |  |
| 1. Health* | 962,711 | 401,514 | 1,596,776 | 409,230 | 52,269 | 1,854,025 | 1,536,202 | 362,283 | 147,541 | 7,322,551 |
| 2. Emergency* | 65,075 | 38,998 | 140,094 | 14,896 | 2,455 | 170,419 | 125,568 | 16,566 | 6,630 | 580,701 |
| 3. Emergencies | 67,988 | 38,982 | 9,006 | 15,514 | 2,759 | 181,488 | 130,251 | 17,432 | 529 | 463,949 |
| 4. 2 OR 3 | 68,002 | 40,156 | 144,886 | 15,515 | 2,759 | 181,521 | 130,253 | 17,432 | 6,920 | 607,444 |
| 5. Hospital* | 327,352 | 158,387 | 718,117 | 86,582 | 5,983 | 775,026 | 558,941 | 107,568 | 48,102 | 2,786,058 |
| 6. Ambulance* | 2,274 | 1,933 | 5,574 | 476 | 94 | 6,449 | 4,535 | 932 | 1,620 | 23,887 |
| 7. Primary Care | 54,058 | 47,336 | 88,423 | 24,646 | 2,554 | 145,623 | 145,478 | 14,794 | 16,858 | 539,770 |
| 8. 1 OR 4 OR 5 OR 6 OR 7 | 1,387,930 | 582,230 | 2,269,869 | 489,148 | 58,501 | 2,605,731 | 432,845 | 431,676 | 187,735 | 8,445,665 |
| 9. Europe* | 247,031 | 23,018 | 170,769 | 33,406 | 55,192 | 540,720 | 26,983 | 56,390 | 9,927 | 1,163,436 |
| 10. Eastern Europe* | 9,579 | 721 | 3,859 | 1,288 | 4,837 | 28,452 | 25,772 | 1,969 | 364 | 76,841 |
| 11. Migrant* | 18,161 | 2,197 | 9,374 | 4,946 | 9,128 | 30,340 | 32,421 | 1,849 | 592 | 109,008 |
| 12. Immigrant* | 25,068 | 5,213 | 15,251 | 12,956 | 9,573 | 35,187 | 53,877 | 5,348 | 492 | 162,965 |
| 13. 11 OR 12 | 40,923 | 7,080 | 23,593 | 17,074 | 17,394 | 62,246 | 2,055,967 | 6,981 | 1,032 | 2,232,290 |
| **14. 8 AND 9 AND 13** | **786**** | **312**** | **1,062** | **343**** | **132** | **1,219** | **1,392** | **59**** | **100**** | **5,192** |
| **15. 8 AND 10 AND 13** | **110**** | **58**** | **176** | **56**** | **17** | **214** | **224** | **12**** | **20**** | **849** |
| **Total** | **808**** | **348**** | **1,238** | **285**** | **149** | **1,433** | **1,616** | **55***** | **92***** | **5,997** |

^**Before exact duplicates removed.^

^***After exact duplicates removed.^
