# Supplementary material for: Understanding how Eastern European migrants use and experience UK health services: a systematic scoping review
Source: BMC Health Serv Res. 2020 Mar 6;20:173. doi: 10.1186/s12913-020-4987-z (PMC7059702; doi:10.1186/s12913-020-4987-z)
Supplement: Supplementary file 2 — Additional file 2: Table S2. Included publications. [file 12913_2020_4987_MOESM2_ESM.docx]

**Table S2** **Included publications**

| **Author(s) & Year** | **Aims & objectives** | **Population** | **Methods** | **Key Results** | **Discussion** | **Limitations** |
| --- | --- | --- | --- | --- | --- | --- |
| Bray, J., et al. (2010) Obstetric Care of New European Migrants in Scotland: an audit of antenatal care, obstetric outcomes and communication | To audit obstetric care standards in Lothian for new migrants and recommend service improvements. | A8 migrant mothers in Lothian, Scotland. | Retrospective audit of 114/136 (84%) obstetric case records of new European migrants giving birth in Lothian hospitals in 2006. Assessment against care standards for antenatal booking, antenatal attendance and need for interpreter. Obstetric outcomes audited against general population. | Interpreter required for 43% of A8 women.  Recording use of interpretation services poor and selective. | Standards for interpretation not met due to underuse, selective use, or under recognition of need for interpretation services.  Common use of informal interpreters, such as family, and planning for interpretation poor.  Poor communication adversely affected care, for example causing increased distress to women in labour, lack of understanding of what was happening and delays in receiving analgesia.  Larger scale research to assess obstetric outcomes of A8 migrants recommended to ensure they are receiving equitable care. Further research should explore cultural differences in antenatal scanning and analgesia use so their needs can be adequately addressed. Mental healthcare of A8 migrants around pregnancy should also be investigated as research evidence suggests poorer health is likely. | Poor documentation on communication. Unclear whether there were omissions in documentation or under-use of interpretation. |
| **Author(s) & Year** | **Aims & objectives** | **Population** | **Methods** | **Key Results** | **Discussion** | **Limitations** |
| Burns (2009) Increased attendances of people of Eastern European  origin at sexual health services in London | To describe service use of migrants from eight central and Eastern European (CEE) countries at two central London genitourinary medicine (GUM) clinics before and after EU accession. | Migrants from eight CEE countries at two central London genitourinary medicine (GUM) clinics before and after EU accession. | KC60 data collected between 1 June 2001 and 30 April 2007. Data refer to new attendances and exclude follow-up appointments. | Of 979 people from CEE countries attending in this year, 44.9% were from Poland, 17.8% from Lithuania, 10.8% from Slovakia, 10.5% from Czech Republic, 7.3% from Hungary, 6.1% from Latvia, 2.2% from Estonia and 0.5% from Slovenia (p=0.634 for gender difference).  Men more likely to have at least one acute STI diagnosed at clinic visit(s): 27.3% (95% CI 26.5% to 28.0%) versus 15.6% (95%CI 15.1% to 16.2%), (p<0.001). However, CEE men just as likely to have an acute STI diagnoses as other men: 29.0% (95% CI 24.5% to 33.8%) versus 27.2% (95% CI 26.5% to 27.9%), age-adjusted OR 1.07 (95% CI 0.85 to 1.33, p=0.578) as were CEE women compared with women from other countries: 14.4% (95% CI 12.6% to 16.4%) versus 15.7% (95%CI 15.1% to 16.3%), age-adjusted OR 0.87 (95% CI 0.74 to 1.02, p=0.090). | Migration favours younger, economically productive and healthier. Also associated with rupture and re-establishment of sexual relationships. Many individuals initially migrate without primary partners. Migration identified as critical factor in high-risk sexual behaviour independent of marital and cohabitation status. All these factors suggest that migrants from CEE at risk of sexual ill health and reproductive morbidity. | Two main limitations. Firstly, does not link behaviour with diagnoses. Secondly, missing data limits reliability. |

| **Author(s) & Year** | **Aims & objectives** | **Population** | **Methods** | **Key Results** | **Discussion** | **Limitations** |
| --- | --- | --- | --- | --- | --- | --- |
| Burns (2011) Sexual and HIV risk behaviour in CEE migrants in London | Study aimed to assess sexual lifestyles and health service needs of CEE migrants in London. | Literate men and women aged over 17 who self-identified as migrants from one of 10 CEE countries. | Community sample recruited in London and web survey was advertised on websites for CEE nationals in UK. Web survey respondents who gave their home post-code outside London excluded. Fieldwork took place over a 9-month period (July 2008-March 2009). Nine fieldworkers who recruited respondents for community sample were native speakers of six of CEE countries.  Self-completed questionnaire fielded using hand-held computers for community sample and a web survey for Internet sample.  Study relied on convenience sampling to generate cost-effective sufficiently robust sample for detailed analysis. Study adopted community and internet sampling strategies to minimise bias. Social mapping exercise prior to recruitment to capture a broad cross-section of CEE migrants in London.  Community sample (n=2,276) and internet sample (n=372) merged to produce CEE population sample (n=2,648). | CEE women more likely to have had new sexual partner(s) in past year if they reported a same sex partner ever (aOR 2.31, 95% CI 1.26 to 4.26), anal or oral sex in past year (aOR 1.48, 95% CI 1.01 to 2.15 and aOR 1.76, 95% CI 1.23 to 2.52 respectively), drinking alcohol more frequently (aOR 1.68, 95% CI 1.02 to 2.80) and a previous STI diagnosis (aOR 1.75, 95% CI 1.13 to 2.73); less likely if married or cohabiting (aOR 0.28, 95% CI 0.20 to 0.38), from A8 (aOR 0.60, 95% CI 0.37 to 0.97), and over 24 years (aOR 0.71, 95% CI 0.51 to 0.98). | Risk of HIV and other STIs may increase as CEE communities become more integrated in UK. High-risk behaviours offset by low prevalence of infections within CEEs. With increasing time, possible that CEE migrants will have sexual partners from more diverse backgrounds, potentially increasing risk of infections.  STI screening opportunities differ in UK compared with many CEE countries, which may impact on STI reports.  Only 31% of reported HIV diagnoses were made in UK. Potentially migrants may be aware of HIV infection but not accessing services in UK, and hence not impacting on national surveillance data yet. | Natsal 2000 data were collected nine years prior to these data. It is possible that frequency of reported behaviours would have changed in British population over time.  Study focused on sexual and reproductive health, but many other aspects to health likely to be relevant to CEE migrants, and subsequently for their host nations. These include high rates of smoking, alcohol consumption and cardiovascular disease. |

| **Author(s) & Year** | **Aims & objectives** | **Population** | **Methods** | **Key Results** | **Discussion** | **Limitations** |
| --- | --- | --- | --- | --- | --- | --- |
| Evans (2011) Central and East European migrant men who have sex  with men: an exploration of sexual risk in the UK | To examine risk for HIV and STIs and use of sexual health services among migrant CEE MSM in UK. | Literate men aged 18 years and over self-identified as migrants from one of 10 CEE countries. Eligibility limited to men reporting at least one male sexual partner in past five years. | SALLEE (Sexual Attitudes and Lifestyles of London’s Eastern Europeans) project based on cross-sectional survey and semi-structured in-depth interviews with CEE men and women living in London.  Anonymous, online questionnaire in 12 languages (10 official CEE languages plus English and Russian).  Men recruited through two popular dating websites for gay men in UK.  Standard statistical tests including Χ^2^, Student’s t test and Mann Whitney U test examined associations between place of residence and background characteristics, risk behaviour and health service use. Logistic regression modelling obtained OR and adjusted OR (aOR) to examine association between unprotected anal intercourse (UAI) with casual partner and background characteristics, commercial sex, recreational drug use and HIV status. Analysis used SPSS version 12.0. | Compared with men living outside London, London men significantly more likely to report paying for sex in UK (15.4% vs 6.8%, p=0.005), had ever been diagnosed with an STI (34.1% vs 22.5%, p=0.003) and had taken recreational drugs in past year (41.5% vs 25.2%, p<0.001).  Also less likely to report sexual mixing (18.6% vs 29.1%, p=0.003).  Longer residency in UK, less likely to report assortative sexual mixing (14.9-33.6-%, p<0.001) and more likely to report recreational drug use in past year (31.4-41.4%, p=0.005).  Ever being diagnosed with an STI increased with longer residency for men in UK, 27.6% of men who had been in UK for up to one year to 37.2% of men who had been in UK for at least five years (p=0.035). | Barriers to HIV testing in CEE countries may mean that HIV more likely to remain undiagnosed among MSM in CEE countries compared to UK, but high prevalence of HIV among men who have sex with men (MSM) in UK likely to increase exposure to infection when CEE men have partnerships with men who are not from their home country. | Recruiting MSM through gay websites more likely to include men with higher sexual risk behaviour than in general MSM population.  Relatively small sample size did not provide sufficient statistical power to analyse data at country level. Not only are data on nationality of CEE migrants to UK incomplete, but they may not reflect distribution of CEE MSM. Study limited by reliance on self-reporting and no biological samples. |

| **Author(s) & Year** | **Aims & objectives** | **Population** | **Methods** | **Key Results** | **Discussion** | **Limitations** |
| --- | --- | --- | --- | --- | --- | --- |
| Evans (2011) Factors associated with genitourinary medicine clinic  attendance and sexually transmitted infection diagnosis  among central and east European migrants in London | To examine factors associated with genitourinary medicine (GUM) clinic attendance and STI diagnosis among CEE migrants in London. | Eligible respondents were literate adults who were born or spent formative years in one of those countries and self-identified as migrants one of 10 CEE countries.  Data analysed refer to new attendances; GUM clinic attendees who were known to be HIV positive before visit excluded, as were follow-up GUM clinic appointments unless they resulted in new STI diagnosis. | SALLEE GUM sample recruited from two central London GUM clinics from 1 July 2008 to 31 March 2009. Undertaken by two researchers, one native Polish & one native Lithuanian speaker. Nine fieldworkers involved in community sample recruitment native speakers of six CEE languages.  Self-completed questionnaire available in 12 languages (10 CEE languages plus English and Russian).  Questionnaire focused on sexual risk behaviour, including sexual practices, numbers of partnerships, using condoms, paying for sex, STI and using sexual health services.  Routinely collected data on all attendances of patients from CEE countries and elsewhere at same two GUM clinics analysed for same period.    Standard statistical tests such as Χ^2^ and Student t test examined associations between factors. Logistic regression modelling was used to obtain OR and adjusted OR. Analysis was performed using SPSS version 12.0. | One GUM clinic runs sessions specifically for female sex workers (FSW). Among 295 patients attending these sessions, 50.2% (n=148) from a CEE country, compared with 6.1% of female patients attending all other GUM clinic sessions. Majority of CEE women attending sessions for FSW Romanian (44.6%), Lithuanian (25.7%) or Polish (10.8%).  Overall, male GUM clinic patients more likely than women to have at least one STI diagnoses during visits (18.5% vs 13.3%, p<0.001). CEE women slightly less likely to be diagnosed with STI than women from UK or elsewhere (11.0% vs 13.6%, p=0.032).  SALLEE GUM clinic respondents more likely than community respondents to report at least one homosexual partner in past five years and difference particularly marked among men (54.3% vs 1.8%, p<0.001). | Key factors associated with having new STI diagnosis among CEE patients were gender and age.  People under 25 years-old disproportionately affected by STI. Previous STI reported by 40% of SALLEE GUM clinic respondents.  GUM services may not be reaching all CEE men at risk for STI. | Study did not link data from cross-sectional survey to clinic records, therefore not possible to examine association between reported sexual behaviour and new STI diagnosis. Some data missing on nationality. CEE migrants come from 10 different countries across an area of wide regional variation, but small sample size did not provide sufficient statistical power to analyse behavioural data at country level. |

| **Author(s) & Year** | **Aims & objectives** | **Population** | **Methods** | **Key Results** | **Discussion** | **Limitations** |
| --- | --- | --- | --- | --- | --- | --- |
| Goodwin, R., et al. (2013) Perceived changes in health  and interactions with ‘the paracetamol force’’: a multimethod study | To examine how Polish migrants evaluate changes in their health in their new country and their interactions with NHS in UK. | Polish immigrants in UK, who at first data sweep were living in Britain up to three months and intended to stay in UK for a minimum of two years. | Quantitative data on 172 participants who took part in all three data waves.  Qualitative data collected first from in-depth interviews with 30 respondents who had been in UK for about six months (Phase 1: 18 women, 12 men, aged between 20 and 59 years, M age 30.4 years [SD = 9.6). Interviews repeated one year later with 25 original respondents (Phase 2: 16 women, nine men). Respondents purposively selected from questionnaire data to represent different gender, age, and educational groups; 30 out of 60 participants approached agreed to participate. | Quantitative survey showed most participants, given no financial barriers, would return to Poland for medical treatment. Several factors influence this choice: urgency of situation, existence (or absence) of language barrier, availability of health insurance at work in UK, repercussions if they had to travel back to Poland, and availability of support networks in UK.  Poles felt medical checkups in UK less detailed than in Poland. They also felt that GPs in Poland more experienced and competent than UK counterparts. | Interviewees said economic pressures inform decisions about treatment options.  English language fluency important factor in health access. May impact on pro-British attitudes and support from British social networks.  Poles criticised own health services, so surprising many wanted to return home for treatment. Experiences of health treatment influenced by expectations about treatment at least partly derived from practices back home.  Need better understanding of pre-migration health systems and continuing transnational health options when attempting to model and promote migrant health behaviour. | Questionnaire data relied on self-reports from Internet. Such reports risk common error variance inflating correlations. Overburdening respondents with long questionnaires often increases non-response. |
| **Author(s) & Year** | **Aims & objectives** | **Population** | **Methods** | **Key Results** | **Discussion** | **Limitations** |
| Ignaszak-Szczepaniak, M. (2009) Reasons for visiting Polish primary care practices by patients aged 18–44 years:  the largest emigrating age group | To analyse reasons for patients’ visits to GPs in Poland, give sample of possible reasons why Polish patients living abroad may make appointments with GPs in other countries. | 12,535 patients aged 18–44 years registered with GPs of ‘Medicus’ Practice, in and around Chodziez, Western Poland. | Data collected from June 2005 to May 2006. Patient’s age defined at beginning of data collection. Patients categorised into three age subgroups (18–24, 25–34 and 35–44 years) in which spectrum of health problems analysed with ICD 10 codes. | Women more likely to visit GP than men, especially those aged 35-44 years. Men of same age saw GP less frequently. Among men, 25–34 year-olds have highest attendance. Women aged 25–34 years less likely to visit GP.  Most common medical problems were hypertension for those 25 years and older, and coronary artery disease for those 35 years and older. Patients aged 25–35 years visited GPs more often for digestive tract disorders: 8.2% vs. 3.6% and 6.5% for those aged 18–24 and 35–44 years, respectively, without differences between women and men. | Only 69% of Polish women aged 25–34 years required consultations, whereas attendance of British women at same age higher.  Unlike UK, perinatal care in Poland given by obstetricians/gynaecologists outside GP practice.  In UK, many consultations by 18- to 24-year olds concerned with sexual health, but consultations not performed routinely by Polish GPs. Women aged 35-44 most likely to visit GP. | Age–gender profile of population cannot be same as age–gender profile of Polish immigrants. Study does not analyse health behaviours of Polish people living abroad.  Polish people’s health habits can change while moving to UK. Changing place of residence may have various effects on frequency of visits to doctor in other countries. |

| **Author(s) & Year** | **Aims & objectives** | **Population** | **Methods** | **Key Results** | **Discussion** | **Limitations** |
| --- | --- | --- | --- | --- | --- | --- |
| Jackowska, M. (2013) Cervical screening among migrant women: a qualitative study of Polish, Slovak and Romanian women in London, UK | To explore awareness of and participation in cervical screening services in women from Poland, Slovakia and Romania living in London, UK. | Women from Poland, Slovakia and Romania living in London. | Study 1 – expert interviews. NHS and other professionals with knowledge of cervical screening among CEE migrants.  Study 2 – focus groups with women. Five focus groups with Polish, Slovak and Romanian women living in London.  Study 3 – interviews with women. Semi-structured interviews to better understand women’s screening participation. | Study 1 - Some health professionals felt that CEE women unaware of recommended screening frequency or age of first screening test.  Study 2 - Women impressed that screening in England free and invitations sent automatically, but many surprised test undertaken by nurse or GP, rather than gynaecologist like in countries of origin.  Study 3 - All these women had been in UK for at least 2 years and tended to be positive about NHS. | Although some women fully engaged in NHS Cervical Screening Programme (NHSCSP), many used screening services in home countries either instead of, as well as NHS.  Although only a few women cited language as barrier to screening for themselves, many believed that poor English and lack of translators was barrier for others.  Romanian women had very negative views about Romanian healthcare, and mainly used NHS for screening, whereas young Slovak women more likely to return to Slovakia for screening and other aspects of health care.  Need to increase healthcare professional cultural competence, to minimise cultural barriers to screening. Need to ensure language not a barrier to participation. | Participant recruitment challenging.  Only able to include Slovak women in their twenties. Most of them had only been in UK for a short time and majority were university educated. Limits generalisability.  Women of all three nationalities tended to be in twenties and thirties so few conclusions can be drawn about older women.  All women recruited in London. Because screening participation and barriers may be different in other areas of UK. |

| **Author(s) & Year** | **Aims & objectives** | **Population** | **Methods** | **Key Results** | **Discussion** | **Limitations** |
| --- | --- | --- | --- | --- | --- | --- |
| Leaman, A. M., et al. (2006) Use of the emergency department by Polish migrant workers | To analyse attendances by patients of Polish origin presenting at Princess Royal Hospital, Telford with GP conditions and extent to which this related to language difficulties. | Patients of Polish origin who had attended ED at Princess Royal Hospital, Telford. | Patients identified using novel computer program assigning country of origin according to a patient’s given and family names. ED’s records of past six years screened, up to and including 2005. Patients of Polish origin further subdivided into those registered with a GP and those not. Among unregistered Polish patients who attended in 2005, convenience sample of 90 further analysed.  Appropriateness of attendance assessed by experienced practitioners in emergency medicine and general practice, using agreed criteria. | In first 18 months following EU expansion, increase in patients of Polish origin attending ED at Princess Royal Hospital, Telford of over 150% from an average of 134 (2000–2003) to 357 in 2005. Of these 357 patients, 152 (43%) not registered with GP. Departmental average for unregistered patients was 7.4%.  Convenience sample of 90 unregistered Polish patients who attended in 2005 showed communication difficulties in at least 47 cases (52%) and that 35 (39%) presented with conditions that could have been treated by GP. | Many these attendances complicated by communication difficulties, and could have been dealt with by GP.  Communication problems can jeopardise good care and can be associated with delays while interpretation facilities are arranged. Usually best if patients who do not speak English present to ED with someone who can interpret on their behalf.  When migrant workers apply to work in UK, should be given information in own language about how to use NHS. | Small sample size limits statistical power and extent to which findings can be generalised more widely. |

| **Author(s) & Year** | | **Aims & objectives** | | **Population** | **Methods** | **Key Results** | **Discussion** | **Limitations** |
| --- | --- | --- | --- | --- | --- | --- | --- | --- |
| Main, I. (2014) Medical Travels of Polish Female Migrants in Europe | | To profile medical travel of Polish women migrants in London, Barcelona, and Berlin. | | Polish women migrants based on study conducted from 2008-2011 on Polish women who migrated to London, Barcelona, and Berlin. | 98 interviews with female migrants and health professionals: 38 in Barcelona, 42 in Berlin, and 18 in London.  Snowball sampling to increase diversity of age, education, length of migration stay, and family situation. | Polish women interviewed in London visited Poland and doctors in Poland more often than counterparts in Barcelona. Polish migrants in Berlin also often visited Poland at least a few times a year, yet hardly ever used specialised health care in Poland.  Polish migrants not only travel to Poland to get medical treatments but also undertake trips to other European countries where they used to live or have access to health care.  Polish migrants in Berlin satisfied with German medical care and able to be referred to a specialist and even consult with another doctor under German health insurance. | Lower cost of flying and availability and frequency of air services was more advantageous for Poles in London than in Barcelona, encouraging visits to Poland more often.  Some migrant women entitled to health care under UK NHS chose to arrange private visits to a doctor in Poland because difficult to get a referral to a specialist in UK.  Another reason for medical travel to visit specialists in Poland is long waiting time for doctor’s appointment in UK.  Lack of language competency another reason for dissatisfaction with local health care and motive to travel to visit doctor in Poland. | Language competency, a common barrier, not addressed. |
| **Author(s) & Year** | **Aims & objectives** | | **Population** | | **Methods** | **Key Results** | **Discussion** | **Limitations** |
| Main, I. (2016) Biomedical practices from a patient perspective:  experiences of Polish female migrants in  Barcelona, Berlin and London | To compare narratives of recent Polish migrants about medical treatments within different medical systems and cultures in Europe. | | Interviewees had lived abroad for at least a year, aged 22-65, with diverse educational and social backgrounds and various family situations.  Majority of interlocutors were women aged 25-40, and their medical experiences most commonly related to reproductive health and paediatric care. Women had diverse health situations, though majority in good health. | | 98 interviews with migrants, healthcare professionals, and representatives of Polish associations in private homes, cafes, and various institutions.  Interviews focused on reasons for and trajectory of migration, access to and experiences with, local and Polish healthcare systems, and subsequent changes in health-related practices and beliefs. Women asked about migratory experiences as well as how they used local and Polish healthcare. Study followed eight women (two in London, three in Berlin, three in Barcelona) over extended period of time, including observations of daily routines related to health and treatment. | Major difference was between healthcare in and outside of Poland. Patients in Barcelona often mentioned that medical personnel provided information accessibly.  Women interviewed in Berlin also expressed strong satisfaction with their contact with local doctors.  In all three localities - London, Barcelona and Berlin – women cited high level of personal culture among medical personnel.  Mothers in Berlin received much support from local healthcare professionals, especially from midwives. Women had access to list of nurses and information about their additional skills, such as languages they spoke. | Relations between patients and doctors an obvious consequence of differences in medical cultures.  Language skills in English, Spanish, Catalan or German languages and access to healthcare system factors influencing health-seeking behaviours and health-related practices. Equally important was understanding local healthcare system structure, of communication patterns, and of differences in position of patient in relation to medical personnel.  Healthcare providers in countries of settlement need to consider different communication and decision-making models prevailing in Poland might influence therapeutic decisions of Polish migrant women. | Language competency, a common barrier, not addressed. |

| **Author(s) & Year** | **Aims & objectives** | **Population** | **Methods** | **Key Results** | **Discussion** | **Limitations** |
| --- | --- | --- | --- | --- | --- | --- |
| Richards, J., et al. (2014) | To investigate maternal and infant health needs within Eastern European populations in Bradford. | Eleven participants in Bradford with significant exposure to Eastern European populations. Comprised of two voluntary workers, four health visitors and five community midwives. All female aged 25-55 years. | Topic guide included health and social issues, and access to services. Pilot interview conducted and questions on emergent themes from interviews incorporated. Interviews at participant’s workplace lasting 20-50 minutes. Interviews audio recorded and transcribed verbatim.  Interviews thematically analysed. Researchers developed framework of codes. Themes emerged from groups of codes. Development of themes deductive.  Data saturation by interview 11. | Some women did not view accessing services as beneficial and others were unaware of maternity services. Some did not know how to access them or that services free.  Predominance of midwives rather than doctors during pregnancy, and less frequent scans, confusing for expectant mothers.  Eastern Europeans attended A&E for primary care. Walk-in services specifically for Eastern Europeans viewed positively.  Many healthcare professionals described shortages in services and specific provision for Eastern European women. | Discrimination, cultural practices regarding age of pregnancy, mobility and disempowerment of women major barriers to healthcare.  Language barriers impeded sensitive consultations and publicising health services. Poor attendance at maternal and child health services may be due to lack of awareness about services and varied expectations.  Targeting information about services in various languages would facilitate direct access to midwives. Regular attendance and consistent interpretation services can strengthen relationships and improve how expectant mothers respond to health promotion messages. | Missing perspective of service users themselves. |

| **Author(s) & Year** | **Aims & objectives** | **Population** | **Methods** | **Key Results** | **Discussion** | **Limitations** |
| --- | --- | --- | --- | --- | --- | --- |
| Sime, D. (2014) ‘I think that Polish doctors are better’: Newly arrived migrant children  and their parents' experiences and views of health services in Scotland | To explore experiences of Eastern European migrant children and their parents of accessing healthcare services after migration. | Service providers from health, education and voluntary sectors.  57 children in focus groups, of whom 48 were Polish. 31 girls and 26 boys, aged 7-16 from range of socio-economic backgrounds.  23 families for case studies. Included 29 children with at least one parent from each family. | Data collection from May 2008–June 2010 across urban, semi urban and rural locations in Scotland.  Started with 19 interviews with service providers, from education, health and voluntary sector. They also recruited newly arrived Eastern European children for focus groups.  After focus groups, 23 in-depth family case studies across several locations in Scotland, including 29 children and at least one parent in each family. Case studies included majority of Polish children (n=13). Other cases involved five Lithuanian, four Slovaks, two Bulgarian and two Romanian children, as well as one Hungarian, one Russian and one Czech family.  Interview data then coded thematically and refined. Two researchers coded every transcript for increased reliability. | Comparisons with provision in homeland often made by parents and children, although no consensus on which aspects of healthcare ‘better’ in UK or country of origin.  Uncertainty over families' entitlement to treatment, differences in provision and approaches to treatment lowered trust in system. Language barrier, often made treatment confusing and visits to health practitioners stressful. Families' limited command of English affected ability to get information on services, experience of interacting with medical practitioners and also ability to follow treatment instructions.  Practitioners commented on ‘unrealistic expectations’ of migrants, on homeland provision, which might focus on more immediate access to health practitioners or access to specialists. | Although, families aware of differences in provision between Scotland and their homeland, seemed to lack full knowledge of entitlements to treatment and of community-based facilities available to them.  Migrant families' limited inter-ethnic social networks in their destination country impacts on their access to information and likely to initially make them trust services less.  Differences between health systems across countries, language barrier, different cultural practices around health and illness and distrust towards care can discourage migrants from making effective use of UK health services. | Although data saturation reached, possible that other newly arrived migrants may have different experiences of health services in Scotland or across UK. |
