# Supplementary material for: Understanding how Eastern European migrants use and experience UK health services: a systematic scoping review
Source: BMC Health Serv Res. 2020 Mar 6;20:173. doi: 10.1186/s12913-020-4987-z (PMC7059702; doi:10.1186/s12913-020-4987-z)
Supplement: Supplementary file 3 — Additional file 3: Table S3. Critical Appraisal Skills Programme (CASP) assessment of the included qualitative studies. [file 12913_2020_4987_MOESM3_ESM.docx]

**Table S3 Critical Appraisal Skills Programme (CASP) assessment of the included qualitative studies**

| **Jackowska (2012) Cervical screening among migrant women: a qualitative study of Polish, Slovak and Romanian women in London, UK** | | |
| --- | --- | --- |
| **Was there clear statement of aims of research?**   - What was goal of research? - Why important? - Relevance? | Yes | - Aims of study to: - (1) Identify different patterns of screening attendance in CEE women living in London using evidence from women themselves and professionals working in field. - (2) Explore awareness about NHSCSP in this group of women. - (3) Explore attitudes to the NHS programme and barriers to participation. - Important because rise in migration to UK from EU accession countries has placed demands on NHS. - Poles may have unmet health care needs, being over-represented in manual work and disproportionately likely to present at EDs with primary care problems. Important as it could reduce engagement with preventive health services, including cervical screening programme. Cervical screening programmes in many CEE countries inadequate and poorly organised, which underlies higher incidence and mortality from cervical cancer. |
| **Is qualitative methodology appropriate?**   - If research seeks to interpret or illuminate actions and/or subjective experiences of research participants. - Is qualitative research right methodology for addressing research goal? | Yes | - Study, through interviews and focus groups explores the experiences of professionals and CEE women themselves. - Semi-structured phone and face-to-face interviews with professionals guided by topic guide developed to address study aims. - Semi-structured interviews to gain more in-depth understanding of women’s screening participation. |
| **Was research design appropriate to address aims of research?**   - If researcher justified research design (e.g. have they discussed how they decided which method to use)? | Yes | - Qualitative approach to understand women’s knowledge of NHS cervical screening services and their cervical screening behaviour, and triangulated their views with those of professionals working in field. |
| **Was recruitment strategy appropriate to aims of research?**   - If researcher explained how participants selected. - If they explained why participants were most appropriate to provide access to type of knowledge sought by study. - If any discussions around recruitment (e.g. why some people chose not to take part). | Yes | - NHS professionals were recruited through Primary Care Research Network – Greater London and researchers’ contacts. Non-NHS professional recruited through organisations working with CEE migrants. Participants recruited through advertisements in shops and newspapers, and through charitable organisations who provide advice to CEE migrants. Snowball sampling recruited their friends and colleagues. - Because research aimed at exploring awareness of NHSCSP among CEE women countries, chose qualitative approach to understand women’s knowledge of NHS cervical screening services and their cervical screening behaviour. - No discussion around why some people chose not to participate. |
| **Was data collected in a way that addressed research issue?**   - If setting for data collection justified. - If it is clear how data collected (e.g. focus group, semi-structured interview etc.). - If researcher justified methods chosen. - If researcher made methods explicit (e.g. for interview method, is there indication of how interviews conducted, or did they use topic guide)? - If methods modified during study. If so, has researcher explained how and why? - If form of data clear (e.g. tape recordings, video material, notes etc.). - If researcher discussed data saturation. | Yes | - Method of data collection justified but not setting for data collection. - Study 1 – interviews with experts; Study 2 – focus groups with women; Study 3 – interviews with women. - Study wanted to address aims through triangulating perspectives of professionals with those of CEE women. - Study 1 - Nine semi-structured phone and two face-to-face interviews, guided by topic guide to address study aims. Participants asked to express opinions about CEE women’s use of NHSCSP. Study 2 - Five focus groups with Polish, Slovak and Romanian women living in London. Focus groups were carried out in participants’ native language structured around topic guide. Polish focus groups carried out by first author in Polish, and Slovak and Romanian groups moderated by native speakers with social science research backgrounds who had been thoroughly briefed about aims of study. Study 3 – interviews with 11 Polish, seven Slovak and two Romanian women. Topic guide similar to that used in focus groups. - No discussion of modification. - All data digitally recorded with participants’ permission, translated where necessary and transcribed in English. |
| **Has relationship between researcher and participants been adequately considered?**   - If researcher critically examined own role, potential bias and influence during formulation of research questions. - Data collection, including sample recruitment and choice of location. - How researcher responded to events during study and whether they considered implications of any changes in research design. | Yes | - Nine semi-structured phone and two face-to-face interviews conducted, guided by a topic guide to address study aims. Study 2 topic guide developed with reference to literature and research questions. Study 3 topic guide similar to that used in Study 2 focus groups. - NHS professionals recruited through Primary Care Research Network – Greater London and researchers’ contacts. Setting chosen because study targeted primary care trusts with large populations of CEE migrants. Five focus groups with Polish, Slovak and Romanian women living in London. - No discussion of design modifications. |
| **Have ethical issues been taken into consideration?**   - If there are sufficient details of how research explained to participants for reader to assess whether ethical standards maintained. - If researcher discussed issues raised by study (e.g. issues around informed consent or confidentiality or how handled effects of study on participants during and after study). - If approval sought from ethics committee. | No | - No discussion of how research explained to participants. - No discussion of informed consent or confidentiality. - Ethical approval granted by University College London (UCL) Research Ethics Committee. |
| **Was data analysis sufficiently rigorous?**   - If there is in-depth description of analysis process. - If thematic analysis used. If so, is it clear how categories/themes derived from data? - Whether researcher explains how data presented selected from original sample to demonstrate analysis process. - If sufficient data presented to support findings. - To what extent contradictory data taken into account. - Whether researcher critically examined own role, potential bias and influence during analysis and selection of data for presentation. | Yes | - Framework analysis, a matrix based approach to qualitative data synthesis. Emerging themes and subthemes identified and used to create thematic framework developed and discussed between two of the authors. - Transcribed data summarised in thematic charts allow data examination either by theme or across participant/group. - Separate analyses carried out for each sub-study, but all analyses informed by issues emerging from other studies. Interpretation of data discussed among all authors. - Quotes illustrated themes from all participant groups. - One practice nurse said: “*People think it’s to pick up cancer and this sort of thing*”. Nevertheless, expert participants felt that most CEE women seemed aware of need for screening, with many having participated in screening in home countries: “*so they may not know why you have screening exactly …but they know they should go*”. - No examination of researcher role, bias and influence during analysis and selection of data for presentation. Interpretation and analysis cross-checked and validated by two authors. |
| **Is there clear statement of findings?**   - If findings explicit. - If there is adequate discussion of evidence both for and against researchers’ arguments. - If researcher discussed credibility of findings (e.g. triangulation, respondent validation, more than one analyst). - If findings discussed in relation to original research question. | Yes | - Findings arranged thematically and are clear. - Participants aware of the importance of cervical screening without necessarily being fully informed about programme, or exact purpose. - Emerging themes and subthemes identified and used to create thematic framework developed and discussed between two authors. - Themes relate to awareness of services and barriers to participation (two key aims). |
| **How valuable is research?**   - If researcher discusses contribution study makes to existing knowledge or understanding e.g. do they consider findings in relation to current practice, policy or relevant research-based literature? - If they identify new areas where research is necessary if researchers discussed whether or how findings can be transferred to other populations or considered other ways research may be used. | | - Highlights areas of potential improvement from provider and user perspectives. Service providers need to do more to help Eastern European women to understand why 3-year screening interval needed. Language and cultural competency still barriers to effective service delivery, which providers to need to address. - Because screening participation and barriers may be different in other areas of UK, future research should address this issue urgently, as large proportion of CEE migrants live outside London. Future work could also usefully examine impact of factors that might affect women’s interactions with NHS, such as having children and being married to UK national. |

| **Main (2014) Medical travels of Polish female migrants in Europe** | | |
| --- | --- | --- |
| **Was there clear statement of aims of research?**   - What was goal of research? - Why important? - Relevance? | No | - Analyses medical travel of Polish women migrants based on study conducted between 2008 and 2011 on Polish women who migrated to London, Barcelona, and Berlin. - Post-2004 accession of Poland to EU accorded their citizens’ rights to freedom of movement, which gave rise to increased migration. - Medical travel one manifestation of Polish citizens having right to freedom of movement. |
| **Is qualitative methodology appropriate?**   - If research seeks to interpret or illuminate actions and/or subjective experiences of research participants. - Is qualitative research right methodology for addressing research goal? | Yes | - Subjective experiences through construction of narratives. - Explorative study of medical travel, and reasons for it, among Polish migrants so qualitative methodology is appropriate. |
| **Was research design appropriate to address aims of research?**   - If researcher justified research design (e.g. have they discussed how they decided which method to use)? | Yes | - Explorative study of medical travel, and reasons for it, among Polish migrants so qualitative methodology is appropriate. |
| **Was recruitment strategy appropriate to aims of research?**   - If researcher explained how participants selected. - If they explained why participants were most appropriate to provide access to type of knowledge sought by study. - If any discussions around recruitment (e.g. why some people chose not to take part). | Yes | - Selection made using snowball and purposive sampling aimed at interviewing diverse groups across ages, education, length of migration stay, and family situation. - Female migrants chosen for interviews had lived abroad for at least a year from diverse educational and social backgrounds and different family situations. Aim to cover range of ages, education, length of migration stay, and family situation. Women also varied in state of health, ranging from very healthy to terminally ill. Interviews conducted only among women because observed that they usually had more experience of medical care, visited doctors and nurses more often, and not only cared for their own health but also for that of their children and husbands/partners. - No discussion of why some women chose not to participate. |
| **Was data collected in a way that addressed research issue?**   - If setting for data collection justified. - If it is clear how data collected (e.g. focus group, semi-structured interview etc.). - If researcher justified methods chosen. - If researcher made methods explicit (e.g. for interview method, is there indication of how interviews conducted, or did they use topic guide)? - If methods modified during study. If so, has researcher explained how and why? - If form of data clear (e.g. tape recordings, video material, notes etc.). - If researcher discussed data saturation. | Yes | - Author was herself a temporary migrant in London, Barcelona and Berlin so was familiar with surroundings. - 98 interviews with female migrants and health professionals: 38 in Barcelona, 42 in Berlin, and   18 in London. Over an extended period spoke to 10 women out of 98 (3 in London, 3 in Barcelona and 4 in Berlin), met their families and friends, and observed daily routines. Interviews in private homes, cafes, and various institutions. Places where migrants often frequent.   - Interviews examined reasons for and trajectories of migration, respondents’ access to and experience with local, Polish, and other health-care systems, and changes to health-related practices and beliefs. Ethnographic study. - No discussion of design modification. - No discussion of form of data. - No discussion of data saturation. |
| **Has relationship between researcher and participants been adequately considered?**   - If researcher critically examined own role, potential bias and influence during formulation of research questions. - Data collection, including sample recruitment and choice of location. - How researcher responded to events during study and whether they considered implications of any changes in research design. | Yes | - Cities chosen for personal reasons and connections. She had undertaken medical travel and had lived in the cities so was familiar with some of the issues under exploration. • - Female migrants chosen for interviews had lived abroad for at least a year from diverse educational and social backgrounds and different family situations. Aim to cover range of ages, education, length of migration stay, and family situation. Women also varied in state of health, ranging from very healthy to terminally ill. Cities chosen for personal reasons and connections. - No discussion of changes in design. |
| **Have ethical issues been taken into consideration?**   - If there are sufficient details of how research explained to participants for reader to assess whether ethical standards maintained. - If researcher discussed issues raised by study (e.g. issues around informed consent or confidentiality or how handled effects of study on participants during and after study). - If approval sought from ethics committee. | No | - No mention of how research explained to participants. - No mention of informed consent or confidentiality. - No mention of ethical approval. |
| **Was data analysis sufficiently rigorous?**   - If there is in-depth description of analysis process. - If thematic analysis used. If so, is it clear how categories/themes derived from data? - Whether researcher explains how data presented selected from original sample to demonstrate analysis process. - If sufficient data presented to support findings. - To what extent contradictory data taken into account. - Whether researcher critically examined own role, potential bias and influence during analysis and selection of data for presentation. | Yes | - Although several interviews with health professionals conducted, analysis of medical travel based on patients’, not professionals’ narratives. - Analysis of narratives to find emerging themes. - Themes represent interpretation of interviews undertaken. - Detailed presentation of themes, but not so much on the analytical process itself. - Multiple quotes within each theme. - Interviewees unhappy with waiting times in UK. Aware that private treatment quicker. But private treatment more expensive than seeking treatment in Poland. Lack of language competency cited as a reason for dissatisfaction with local healthcare although none professed to having limited language skills. - Author familiar with all cities because she herself was temporary migrant in all three. She had undertaken medical travel too so was aware of some issues. |
| **Is there clear statement of findings?**   - If findings explicit. - If there is adequate discussion of evidence both for and against researchers’ arguments. - If researcher discussed credibility of findings (e.g. triangulation, respondent validation, more than one analyst). - If findings discussed in relation to original research question. | Yes | - Findings explicit. - Presented in nuanced way which captures complexity of decision making. - Only author involved in analysis. Not cross-checked by another. - Themes relate to reasons for undertaking medical travel, which is what study aims to address. |
| **How valuable is research?**   - If researcher discusses contribution study makes to existing knowledge or understanding e.g. do they consider findings in relation to current practice, policy or relevant research-based literature? - If they identify new areas where research is necessary if researchers discussed whether or how findings can be transferred to other populations or considered other ways research may be used. | | - Highlights issues in medical travel, which could be useful for research, policy and practice. - Did not identify potential new areas of research. |

| **Main (2016) Biomedical practices from a patient perspective. Experiences of Polish female migrants in Barcelona, Berlin and London** | | |
| --- | --- | --- |
| **Was there clear statement of aims of research?**   - What was goal of research? - Why important? - Relevance? | Yes | - To compare and juxtapose narratives of recent Polish migrants about medical treatments within different medical systems and cultures in Europe. - This case study fills a gap, analysing situation of migrant Polish women who experienced medical pluralism during pregnancy, childbirth, and motherhood in three European cities. - Polish migration to EU important because of EU’s open labour market. |
| **Is qualitative methodology appropriate?**   - If research seeks to interpret or illuminate actions and/or subjective experiences of research participants. - Is qualitative research right methodology for addressing research goal? | Yes | - Author explores stance of migrants on medical treatment, and impact of having more knowledge and experiences about different medical practices because of migration. Study explores subjective experiences of patients both from their perspective and that of health professionals. - Because it is exploring perspectives, qualitative research is appropriate. |
| **Was research design appropriate to address aims of research?**   - If researcher justified research design (e.g. have they discussed how they decided which method to use)? | Yes | - Author was temporary migrant and patient in London, Barcelona and Berlin, which allowed her to explore her own experiences. She could empathise and was aware of some of the issues of her subjects. Ethnographic research conducted among Polish migrants in London, Barcelona and Berlin from 2008 to 2013. 98 interviews with migrants, healthcare professionals, and representatives of Polish associations. |
| **Was recruitment strategy appropriate to aims of research?**   - If researcher explained how participants selected. - If they explained why participants were most appropriate to provide access to type of knowledge sought by study. - If any discussions around recruitment (e.g. why some people chose not to take part). | Yes | - Female migrants chosen for interviews had lived abroad for at least a year, although majority had lived outside of Poland for more than five years; women between 22-65 years-old, with diverse educational and social backgrounds and various family situations. - Participant trajectories similar to that of author. - No mention of why some people did not participate. |
| **Was data collected in a way that addressed research issue?**   - If setting for data collection justified. - If it is clear how data collected (e.g. focus group, semi-structured interview etc.). - If researcher justified methods chosen. - If researcher made methods explicit (e.g. for interview method, is there indication of how interviews conducted, or did they use topic guide)? - If methods modified during study. If so, has researcher explained how and why? - If form of data clear (e.g. tape recordings, video material, notes etc.). - If researcher discussed data saturation. | Yes | - Interviews in private homes, cafes, and various institutions. Places where migrants often frequent. Author was herself a temporary migrant in London, Barcelona and Berlin so was familiar with surroundings. - 98 interviews with female migrants and health professionals: 38 in Barcelona, 42 in Berlin, and 18 in London. Over an extended period spoke to 10 women out of 98 (3 in London, 3 in Barcelona and 4 in Berlin), met their families and friends, and observed daily routines. Interviews in private homes, cafes, and various institutions. Places where migrants often frequent. - Interviews examined reasons for and trajectories of migration, respondents’ access to and experience with local, Polish, and other health-care systems, and changes to health-related practices and beliefs. Ethnographic study. - No discussion of design modification. - No discussion of form of data. - No discussion of data saturation. |
| **Has relationship between researcher and participants been adequately considered?**   - If researcher critically examined own role, potential bias and influence during formulation of research questions. - Data collection, including sample recruitment and choice of location. - How researcher responded to events during study and whether they considered implications of any changes in research design. | Yes | - Cities chosen for personal reasons and connections. She had undertaken medical travel and had lived in the cities so was familiar with some of the issues under exploration. - Female migrants chosen for interviews had lived abroad for at least a year from diverse educational and social backgrounds and different family situations. Aim to cover range of ages, education, length of migration stay, and family situation. Women also varied in state of health, ranging from very healthy to terminally ill. Cities chosen for personal reasons and connections. Interviews in private homes, cafes, and various institutions. Places where migrants often frequent. - No discussion of changes in design. |
| **Have ethical issues been taken into consideration?**   - If there are sufficient details of how research explained to participants for reader to assess whether ethical standards maintained. - If researcher discussed issues raised by study (e.g. issues around informed consent or confidentiality or how handled effects of study on participants during and after study). - If approval sought from ethics committee. | No | - No mention of how research explained to participants. - No mention of informed consent or confidentiality. - No mention of ethical approval. |
| **Was data analysis sufficiently rigorous?**   - If there is in-depth description of analysis process. - If thematic analysis used. If so, is it clear how categories/themes derived from data? - Whether researcher explains how data presented selected from original sample to demonstrate analysis process. - If sufficient data presented to support findings. - To what extent contradictory data taken into account. - Whether researcher critically examined own role, potential bias and influence during analysis and selection of data for presentation. | Yes | • Although several interviews with health professionals conducted, analysis of medical travel based on patients’, not professionals’ narratives.  • Analysis of narratives to find emerging themes.  • Themes represent interpretation of interviews undertaken.  • Detailed presentation of themes, but not so much on the analytical process itself.  • Multiple quotes within each theme.  • Interviewees unhappy with waiting times in UK. Aware that private treatment quicker. But private treatment more expensive than seeking treatment in Poland. Lack of language competency cited as a reason for dissatisfaction with local healthcare although none professed to having limited language skills.  • Author familiar with all cities because she herself was temporary migrant in all three. She had undertaken medical travel too so was aware of some issues. |
| **Is there clear statement of findings?**   - If findings explicit. - If there is adequate discussion of evidence both for and against researchers’ arguments. - If researcher discussed credibility of findings (e.g. triangulation, respondent validation, more than one analyst). - If findings discussed in relation to original research question. | No | - Findings explicit. - Presented in nuanced way which captures complexity of decision making. - Only author involved in analysis. Not cross-checked by another. - Themes relate to reasons for undertaking medical travel, which is what study aims to address. |
| **How valuable is research?**   - If researcher discusses contribution study makes to existing knowledge or understanding e.g. do they consider findings in relation to current practice, policy or relevant research-based literature? - If they identify new areas where research is necessary if researchers have discussed whether or how findings can be transferred to other populations or considered other ways research may be used. | | - Highlights issues in medical travel, which could be useful for research, policy and practice. - Did not identify potential new areas of research. |

| **Richards (2014) Maternal and infant health of Eastern Europeans in Bradford, UK: a qualitative study** | | |
| --- | --- | --- |
| **Was there clear statement of aims of research?**   - What was goal of research? - Why important? - Relevance? | Yes | - To investigate maternal and infant health needs within Eastern European populations in Bradford. - Migrants from Eastern Europe had poor maternal and child health and increased rates of infant mortality. - Bradford has more Slovak speakers than any other local authority in UK and third highest number of Czech speakers. |
| **Is qualitative methodology appropriate?**   - If research seeks to interpret or illuminate actions and/or subjective experiences of research participants. - Is qualitative research right methodology for addressing research goal? | No | - This inductive study aimed to investigate maternal and infant health through exploring the subjective experience of research participants. - Any such investigation amenable to qualitative research. |
| **Was research design appropriate to address aims of research?**   - If researcher justified research design (e.g. have they discussed how they decided which method to use)? | Yes | - Eleven participants chosen by purposive sampling to ensure that interviewees had significant exposure to Eastern European populations. Study through lens of practitioners with experience of working with this population. |
| **Was recruitment strategy appropriate to aims of research?**   - If researcher explained how participants selected. - If they explained why participants were most appropriate to provide access to type of knowledge sought by study. - If any discussions around recruitment (e.g. why some people chose not to take part). | Yes | - Consultant in public health provided introductions to head of health visiting and midwifery at local health services. - Support from three service leads who disseminated information to relevant staff. Potential participants asked to contact researchers directly if they wished to be interviewed. Study through lens of practitioners with experience of working with this population. |
| **Was data collected in a way that addressed research issue?**   - If setting for data collection justified. - If it is clear how data collected (e.g. focus group, semi-structured interview etc.). - If researcher justified methods chosen. - If researcher made methods explicit (e.g. for interview method, is there indication of how interviews conducted, or did they use topic guide)? - If methods modified during study. If so, has researcher explained how and why? - If form of data clear (e.g. tape recordings, video material, notes etc.). - If researcher discussed data saturation. | Yes | - All interviews arranged in advance at a convenient time for researcher and participant. Interviews at participant’s place of work. - Semi-structured interviews. - Topic guide based on findings from literature review. Topics included health and social issues, and access to services. A pilot interview conducted and question on emergent themes from interviews incorporated. - Topic guide based on findings from literature review. Eleven interviews. - No mention of modification in design. - No mention of form of data. - By interview 11 saturation point reached. |
| **Has relationship between researcher and participants been adequately considered?**   - If researcher critically examined own role, potential bias and influence during formulation of research questions. - Data collection, including sample recruitment and choice of location. - How researcher responded to events during study and whether they considered implications of any changes in research design. | Yes | - No mention of potential bias and influence during formulation of research questions. - Eleven participants chosen by purposive sampling to ensure that interviewees had significant exposure to Eastern European populations. Interviews at participant’s place of work. - No changes in design. |
| **Have ethical issues been taken into consideration?**   - If there are sufficient details of how research explained to participants for reader to assess whether ethical standards maintained. - If researcher discussed issues raised by study (e.g. issues around informed consent or confidentiality or how handled effects of study on participants during and after study). - If approval sought from ethics committee. | No | - Support from three service leads who disseminated information to relevant staff. - Written informed consent taken. - No mention of ethical approval. |
| **Was data analysis sufficiently rigorous?**   - If there is in-depth description of analysis process. - If thematic analysis used. If so, is it clear how categories/themes derived from data? - Whether researcher explains how data presented selected from original sample to demonstrate analysis process. - If sufficient data presented to support findings. - To what extent contradictory data taken into account. - Whether researcher critically examined own role, potential bias and influence during analysis and selection of data for presentation. | Yes | - Interviews audio recorded and transcribed verbatim. - Interviews were analysed using thematic analysis. Relevant phrases from transcripts highlighted and assigned a descriptive code. Repeated to ensure intracoder reliability, Framework of codes developed. New codes added, and irrelevant codes discarded, and similar codes combined. Themes emerged from groups of codes, which were relevant in answering study question. Development of themes deductive. Coding from pilot study agreed by second author to ensure intercoder reliability. Consistency between coders established. - Not detailed description of themes but each one reinforced by quotes. - No mention of bias and influence. |
| **Is there clear statement of findings?**   - If findings explicit. - If there is adequate discussion of evidence both for and against researchers’ arguments. - If researcher discussed credibility of findings (e.g. triangulation, respondent validation, more than one analyst). - If findings discussed in relation to original research question. | No | - Findings succinct and clear. - No contradictory evidence presented. - Coding from pilot study agreed by second author to ensure intercoder reliability. Consistency between coders established. - Themes emerged from groups of codes, which were relevant in answering study question. |
| **How valuable is research?**   - If researcher discusses contribution study makes to existing knowledge or understanding e.g. do they consider findings in relation to current practice, policy or relevant research-based literature? - If they identify new areas where research is necessary if researchers discussed whether or how findings can be transferred to other populations or considered other ways research may be used. | | - Better educational opportunities may reduce poverty. Promoting later pregnancy within this population could lead to lower infant mortality. Increased employment of Eastern European healthcare professionals would improve understanding, communication and trust. Targeting information about services in various languages would assist direct access to midwives. Networks, health champions and extended families should be used to disseminate health information. - Study contributes to existing knowledge and understanding but does not identify new areas of research. |

| **Sime (2014) 'I think that Polish doctors are better': Newly arrived migrant children and their parents' experiences and views of health services in Scotland** | | |
| --- | --- | --- |
| **Was there clear statement of aims of research?**   - What was goal of research? - Why important? - Relevance? | Yes | - To look at experiences of children of Eastern European migrant workers arrived in Scotland, focussing on impact of being a new migrant on children's family and peer relationships, factors affecting children's integration and their experiences of accessing services. - Impact of migration on public services is a key issue which frames current debates on immigration. Data on migrants' health scarce, mainly to do with fact that migrants are not homogenous and immigration status not recorded consistently when health services provided. - Evidence suggests Eastern European migrants generally engage with health services less regularly and present increased risk of somatic complaints and illnesses. |
| **Is qualitative methodology appropriate?**   - If research seeks to interpret or illuminate actions and/or subjective experiences of research participants. - Is qualitative research right methodology for addressing research goal? | Yes | - Seeks to illuminate experiences of staff from healthcare, education and voluntary sectors, as well as Eastern European migrant children. - Explorative so qualitative methodology appropriate. |
| **Was research design appropriate to address aims of research?**   - If researcher justified research design (e.g. have they discussed how they decided which method to use)? | Yes | - Focus groups chosen because they allow children to stimulate each other's ideas, and also allow researcher to assess extent to which group share beliefs and experiences. Significant component of research used visual methods, which is felt to be an effective method of working with children. |
| **Was recruitment strategy appropriate to aims of research?**   - If researcher explained how participants selected. - If they explained why participants were most appropriate to provide access to type of knowledge sought by study. - If any discussions around recruitment (e.g. why some people chose not to take part). | Yes | - Empirical data collection between May 2008–June 2010 across range of urban, semi-urban and rural locations in Scotland. Started with 19 interviews with service providers, from education, health and voluntary sector. Then used service providers as ‘gatekeepers’ to recruit newly arrived Eastern European children for focus groups. Following focus groups, 23 in-depth family case studies completed across several locations in Scotland, including 29 children (six pairs of siblings involved) at least one parent in each family. Case studies included majority of Polish children (n=13). Most recruited through focus groups. - Concentrates on perspectives of children and parents, but also adds perspectives of service providers to produce a more rounded study that involves all key stakeholders. - No mention of why some people chose not to take part. |
| **Was data collected in a way that addressed research issue?**   - If setting for data collection justified. - If it is clear how data collected (e.g. focus group, semi-structured interview etc.). - If researcher justified methods chosen. - If researcher made methods explicit (e.g. for interview method, is there indication of how interviews conducted, or did they use topic guide)? - If methods modified during study. If so, has researcher explained how and why? - If form of data clear (e.g. tape recordings, video material, notes etc.). - If researcher discussed data saturation. | Yes | - Range of different locations to capture balance as well as involvement of key stakeholders spanning several sectors. - 19 interviews with service providers and 57 children took part in focus groups. Following focus groups, 23 in-depth family case studies completed across several locations in Scotland, including 29 children (six pairs of siblings involved) and at least one parent in each family. - To get in-depth perspectives of all key stakeholders. - Focus groups conducted either in children's own language (for Polish and Romanian), or with interpreters present for other groups. Children encouraged to discuss experiences of a ‘typical migrant family’. - Although data saturation reached, possible that other newly arrived migrants may have different experiences of health services in Scotland or across UK, given differences in attitudes to migration across UK and approaches to health service delivery. - No mention of modification in research design. |
| **Has relationship between researcher and participants been adequately considered?**   - If researcher critically examined own role, potential bias and influence during formulation of research questions. - Data collection, including sample recruitment and choice of location. - How researcher responded to events during study and whether they considered implications of any changes in research design. | Yes | - Informed by concepts of social capital and social networks, collection of migrant families' experiences of health and their health practices was guided by two main questions: What are Eastern European migrant children's and their parents' views and experiences of health service provision in Scotland? and What are main barriers encountered by them when engaging with health services and how do migrants overcome these? - As migrants navigate various networks, interested in examining how practices around health and healthcare shaped by ongoing engagement with people and services in various fields, including through ties maintained with family members and services in homeland. Because of this interest in connections between providers and migrants, study used service providers as ‘gatekeepers’ to recruit newly arrived Eastern European children for focus groups. - No mention of changes in research design. |
| **Have ethical issues been taken into consideration?**   - If there are sufficient details of how research explained to participants for reader to assess whether ethical standards maintained. - If researcher discussed issues raised by study (e.g. issues around informed consent or confidentiality or how handled effects of study on participants during and after study). - If approval sought from ethics committee. | Yes | - Children informed of study through translated leaflets, which outlined their expected participation. - They signed consent form. - No mention of ethical approval. |
| **Was data analysis sufficiently rigorous?**   - If there is in-depth description of analysis process. - If thematic analysis used. If so, is it clear how categories/themes derived from data? - Whether researcher explains how data presented selected from original sample to demonstrate analysis process. - If sufficient data presented to support findings. - To what extent contradictory data taken into account. - Whether researcher critically examined own role, potential bias and influence during analysis and selection of data for presentation. | Yes | - All interviews, focus groups and case study visits tape recorded with participants' consent, translated into English when necessary, transcribed and analysed using grid analysis and thematic coding. - Overview thematic grid produced to map out descriptive summaries of issues emerging, leading to range of common key themes to develop across dataset. Relevant sections of data from transcripts assigned appropriate thematic codes and refined sub-categories emerged and allocated to text in transcripts. NVivo7 to facilitate organising and classifying data. Two researchers coded every transcript for increased reliability. - Two key themes – migrant children’s perceptions of health service and barriers to use. Under barriers to use, four sub-themes – lack of adequate information; differences in provision; language barrier; and transnational use of health services. - Findings not nuanced; consistent thread throughout. - Theories of social capital influenced analysis and thematic content. |
| **Is there clear statement of findings?**   - If findings explicit. - If there is adequate discussion of evidence both for and against researchers’ arguments. - If researcher discussed credibility of findings (e.g. triangulation, respondent validation, more than one analyst). - If findings discussed in relation to original research question. | Yes | - Findings explicit and succinct. - Findings not nuanced, therefore only evidence in support of researchers’ arguments. - NVivo7 to facilitate process of organising and classifying data and two researchers coded every transcript for increased reliability. |
| **How valuable is research?**   - If researcher discusses contribution study makes to existing knowledge or understanding e.g. do they consider findings in relation to current practice, policy or relevant research-based literature? - If they identify new areas where research is necessary if researchers have discussed whether or how findings can be transferred to other populations or considered other ways research may be used. | | - Findings also revealed complex and varied attitudes and approaches to engaging with services, which were often more sophisticated than service providers might expect. Suggests that migrants' needs and beliefs about what health services should provide and how they should operate need full consideration to inform healthcare policy and practitioners' behaviours when engaging with newly migrated patients. Healthcare providers must be sensitive to personal factors which affect migrant children and their families' use of healthcare and work with them to tailor provision. - Evidence that UK health services might consider migrants a homogenous group and not take into account their diverse needs, socio-economic backgrounds, experiences and attitudes to engagement with health services. Practitioners need to make migrants' engagement with services positive and take their past experiences and views into account when considering improvements in delivery. Fuller, further interrogation of nature of migrants' experiences and practices around health and their expectations of health care post-migration needed. |
