# Supplementary material for: Understanding how Eastern European migrants use and experience UK health services: a systematic scoping review
Source: BMC Health Serv Res. 2020 Mar 6;20:173. doi: 10.1186/s12913-020-4987-z (PMC7059702; doi:10.1186/s12913-020-4987-z)
Supplement: Supplementary file 4 — Additional file 4: Table S4. Joanna Briggs Institute (JBI) checklist for analytical cross-sectional studies. [file 12913_2020_4987_MOESM4_ESM.docx]

**Table S4 Joanna Briggs Institute (JBI) checklist for analytical cross-sectional studies**

| **Bray (2010) Obstetric care of new European migrants in Scotland: an audit of antenatal care, obstetric outcomes and communication** | | |
| --- | --- | --- |
| **Were inclusion criteria in sample clearly defined?**   - Authors should provide clear inclusion and exclusion criteria developed prior to recruiting study participants. Inclusion/exclusion criteria should be specified with sufficient detail and all necessary information critical to study. | Yes | - Retrospective audit of maternity case records for mothers born in A8 country who gave birth in Lothian hospital in 2006. |
| **Were study subjects and setting described in detail?**   - Study sample should be described in sufficient detail so that other researchers can determine if comparable to population of interest to them. Authors should provide clear description of population from which study participants selected or recruited, including demographics, location, and time period. | Yes | - Mothers born in A8 country who gave birth in a Lothian hospital in 2006. |
| **Was exposure measured in valid and reliable way?**   - Study should clearly describe method of measurement of exposure. Assessing validity requires that 'gold standard' is available to which measure can be compared. Validity of exposure measurement usually relates to whether current measure is appropriate or whether measure of past exposure needed. - Reliability refers to processes included in an epidemiological study to check repeatability of measurements of exposures. These usually include intra-observer reliability and inter- observer reliability. | Yes | - Findings for A8 population audited against NHS Lothian care standards. Available for antenatal booking date, number of antenatal appointments attended and interpretation and minimum target for compliance with these standards was taken as 80%. Obstetric outcomes for A8 population audited against outcomes for Lothian population where possible. Comparison dataset obtained from Royal Infirmary of Edinburgh Maternity Management System. |
| **Were objective, standard criteria used for measurement of condition?**   - Useful to determine if patients included in study based on either specified diagnosis or definition. More likely to decrease risk of bias. Characteristics another useful approach to matching groups, and studies that did not use specified diagnostic methods or definitions should provide evidence on matching by key characteristics. | No | - Mothers born in A8 country who gave birth in a Lothian hospital in 2006. No specific condition or disease under consideration. |
| **Were confounding factors identified?**   - Confounding occurred where estimated intervention exposure effect biased by presence of some difference between comparison groups (apart from exposure investigated/of interest). Typical confounders include baseline characteristics, prognostic factors, or concomitant exposures (e.g. smoking). Confounder is difference between comparison groups and it influences direction of study results. A high quality study at level of cohort design will identify potential confounders and measure them (where possible). Difficult for studies where behavioural, attitudinal or lifestyle factors may impact on results. | No | - Obstetric outcomes not significantly different to general population but study small and no adjustment can be made for confounding factors, such as age, parity and socio-economic status. |
| **Were strategies to deal with confounding factors stated?**   - Strategies to deal with effects of confounding factors may be dealt within study design or in data analysis. By matching or stratifying sampling of participants, effects of confounding factors can be adjusted for. When dealing adjustment in data analysis, assess statistics used in study. Most will be some form of multivariate regression analysis to account for confounding factors measured. | No | - No regression analysis performed. |
| **Were outcomes measured in valid and reliable way?**   - Read methods section of paper. If for e.g. lung cancer is assessed based on existing definitions or diagnostic criteria, then answer likely to be yes. If lung cancer assessed using observer reported, or self‐reported scales, risk of over‐ or under-reporting increased, and objectivity compromised. Importantly, determine if measurement tools used validated instrument which has significant impact on outcome assessment validity. - Having established objectivity of outcome measurement (e.g. lung cancer) instrument, it’s important to establish how measurement conducted. Were those involved in collecting data trained or educated in use of instrument/s? (e.g. radiographers). If more than one data collector, similar in terms of level of education, clinical or research experience, or level of responsibility in piece of research being appraised? | Yes | - Retrospective audit of maternity case records for mothers born in A8 country who gave birth in Lothian hospital in 2006. Information on numbers of women giving birth in Scotland and mother’s place of birth obtained from General Register Office (GRO) birth registration records. Names of eligible mothers matched with hospital birth records which were examined by research team. |
| **Was appropriate statistical analysis used?**   - As with any consideration of statistical analysis, should consider whether more appropriate alternate statistical method could have been used. Methods section of cohort studies should be detailed enough for reviewers to identify which analytical techniques used (in particular, regression or stratification) and how specific confounders measured. - For studies utilising regression analysis, useful to identify if study identified which variables included and how related to outcome. If stratification was analytical approach used, were strata of analysis defined by specified variables? Also important to assess appropriateness of analytical strategy in terms of assumptions associated with approach as differing methods of analysis based on differing assumptions about data and how it will respond. | No | - Regression could not be used for such a small sample. |

| **Burns (2009) Increased attendances of people of Eastern European origin at sexual health services in London** | | |
| --- | --- | --- |
| **Were inclusion criteria in sample clearly defined?**   - Authors should provide clear inclusion and exclusion criteria developed prior to recruiting study participants. Inclusion/exclusion criteria should be specified with sufficient detail and all necessary information critical to study. | Yes | - People presenting to Genito-Urinary Medicine (GUM) clinic in London with STI. Compared people from eight CEE countries with people born elsewhere. Study not cohort study because it does not follow a group of people from start to end. Cross-sectional because only focuses on new cases, not follow-ups from original. HIV positive patients excluded. |
| **Were study subjects and setting described in detail?**   - Study sample should be described in sufficient detail so that other researchers can determine if comparable to population of interest to them. Authors should provide clear description of population from which study participants selected or recruited, including demographics, location, and time period. | Yes | - 102,604 people attended clinics at least once in study period. They generated 154,964 visits from people with known country of birth. 5,805 (3.8%, 95% CI 3.7% to 3.9%) made by people born in CEE country. Data collected from 1 June 2001-30 April 2007*.* |
| **Was exposure measured in valid and reliable way?**   - Study should clearly describe method of measurement of exposure. Assessing validity requires that 'gold standard' is available to which measure can be compared. Validity of exposure measurement usually relates to whether current measure is appropriate or whether measure of past exposure needed. - Reliability refers to processes included in an epidemiological study to check repeatability of measurements of exposures. These usually include intra-observer reliability and inter- observer reliability. | No | - Focused on increased attendances, not risk taking behaviour that gives rise to STIs that they present with. |
| **Were objective, standard criteria used for measurement of condition?**   - Useful to determine if patients included in study based on either specified diagnosis or definition. More likely to decrease risk of bias. Characteristics another useful approach to matching groups, and studies that did not use specified diagnostic methods or definitions should provide evidence on matching by key characteristics. | Yes | - Participants eligible if presenting to GUM clinic with an STI. |
| **Were confounding factors identified?**   - Confounding occurred where estimated intervention exposure effect biased by presence of some difference between comparison groups (apart from exposure investigated/of interest). Typical confounders include baseline characteristics, prognostic factors, or concomitant exposures (e.g. smoking). Confounder is difference between comparison groups and it influences direction of study results. A high quality study at level of cohort design will identify potential confounders and measure them (where possible). Difficult for studies where behavioural, attitudinal or lifestyle factors may impact on results. | Yes | - Confounding factors include age, gender, employment status. Tables 3 (men) & 4 (women) included age-adjusted odds ratios. |
| **Were strategies to deal with confounding factors stated?**   - Strategies to deal with effects of confounding factors may be dealt within study design or in data analysis. By matching or stratifying sampling of participants, effects of confounding factors can be adjusted for. When dealing adjustment in data analysis, assess statistics used in study. Most will be some form of multivariate regression analysis to account for confounding factors measured. | Yes | - Tables 3 (men) & 4 (women) include age-adjusted odds ratios but not for other confounding factors. Mann-Whitney test used to test for age differences. |
| **Were outcomes measured in valid and reliable way?**   - Read methods section of paper. If for e.g. lung cancer is assessed based on existing definitions or diagnostic criteria, then answer likely to be yes. If lung cancer assessed using observer reported, or self‐reported scales, risk of over‐ or under-reporting increased, and objectivity compromised. Importantly, determine if measurement tools used validated instrument which has significant impact on outcome assessment validity. - Having established objectivity of outcome measurement (e.g. lung cancer) instrument, it’s important to establish how measurement conducted. Were those involved in collecting data trained or educated in use of instrument/s? (e.g. radiographers). If more than one data collector, similar in terms of level of education, clinical or research experience, or level of responsibility in piece of research being appraised? | Yes | - Outcome is presence of STI and attendance at GUM clinic. Binary logistic regression to obtain odds ratios (OR) to compare recording of any acute STI as well as specific acute STI for CEE attendees in contrast to attendees from other countries (including UK), as well as in contrast to attendees born in or from either UK or Ireland. Comparison is made between individuals born in CEE countries and those born elsewhere. - Unclear who collected and entered data and what their expertise was. |
| **Was appropriate statistical analysis used?**   - As with any consideration of statistical analysis, should consider whether more appropriate alternate statistical method could have been used. Methods section of cohort studies should be detailed enough for reviewers to identify which analytical techniques used (in particular, regression or stratification) and how specific confounders measured. - For studies utilising regression analysis, useful to identify if study identified which variables included and how related to outcome. If stratification was analytical approach used, were strata of analysis defined by specified variables? Also important to assess appropriateness of analytical strategy in terms of assumptions associated with approach as differing methods of analysis based on differing assumptions about data and how it will respond. | Yes | - Mann–Whitney tested for differences in age of patients because of non-normal age distribution. Binary logistic regression established odds ratios (OR) to compare recording any acute STI as well as specific acute STI for attendees from CEE countries in contrast to attendees from other countries (including UK), as well as in contrast to attendees born in or from either UK or Ireland. No alternative statistical analysis discussed. - Age adjusted differences in age of attendees from CEE countries versus other countries. Linear regression trend analysis. |

| **Burns (2011) Sexual and HIV risk behaviour in Central and Eastern European migrants in London** | | |
| --- | --- | --- |
| **Were inclusion criteria in sample clearly defined?**   - Authors should provide clear inclusion and exclusion criteria developed prior to recruiting study participants. Inclusion/exclusion criteria should be specified with sufficient detail and all necessary information critical to study. | Yes | - Eligible participants were literate men and women aged 17+ who self-identified as CEE migrants. |
| **Were study subjects and setting described in detail?**   - Study sample should be described in sufficient detail so that other researchers can determine if comparable to population of interest to them. Authors should provide clear description of population from which study participants selected or recruited, including demographics, location, and time period. | Yes | - Eligible participants were literate men and women aged 17+ who self-identified as CEE migrants. No adequate sampling frame of new migrant population from which to draw probability sample of CEE nationals. Convenience sampling. Social mapping exercise before sample recruited. Locations chosen to capture broad cross section of CEE migrants in London. |
| **Was exposure measured in valid and reliable way?**   - Study should clearly describe method of measurement of exposure. Assessing validity requires that 'gold standard' is available to which measure can be compared. Validity of exposure measurement usually relates to whether current measure is appropriate or whether measure of past exposure needed. - Reliability refers to processes included in an epidemiological study to check repeatability of measurements of exposures. These usually include intra-observer reliability and inter- observer reliability. | Yes | - Partners or sexual partners defined as people who have had sex together or whether just once, or a few times, or as regular partners, or as married partners. - Nearly half (44.9%) of men and 29.1% of women had formed new heterosexual partnership over past year. Mean number of new partners declined with increasing age for men. - Risk factors for HIV in respondents who reported being HIV positive were: previous injecting drug use (n=2), sex between men (n=1), six (21%) had ever paid for sex but none reported having been paid for sex, and eight (28%) reported a previous STI diagnosis. |
| **Were objective, standard criteria used for measurement of condition?**   - Useful to determine if patients included in study based on either specified diagnosis or definition. More likely to decrease risk of bias. Characteristics another useful approach to matching groups, and studies that did not use specified diagnostic methods or definitions should provide evidence on matching by key characteristics. | Yes | - Survey of sexual behaviour conducted among CEE migrants attending two central London GUM clinics. Links to Burns (2009), Evans (2011) and Evans (2011) studies reported on by Del Amo (2011) editorial. |
| **Were confounding factors identified?**   - Confounding occurred where estimated intervention exposure effect biased by presence of some difference between comparison groups (apart from exposure investigated/of interest). Typical confounders include baseline characteristics, prognostic factors, or concomitant exposures (e.g. smoking). Confounder is difference between comparison groups and it influences direction of study results. A high quality study at level of cohort design will identify potential confounders and measure them (where possible). Difficult for studies where behavioural, attitudinal or lifestyle factors may impact on results. | Yes | - Logistic regression to obtain adjusted odds ratios to control for variation by age, gender, marital status and education. Overall project compares presentation at GUM clinics with STIs between CEE migrants and those born elsewhere. Table 1 shows distribution of partners over five years and past year by gender and age group. Table 3 shows prevalence of sexual behaviours and drug use by gender and region of origin. |
| **Were strategies to deal with confounding factors stated?**   - Strategies to deal with effects of confounding factors may be dealt within study design or in data analysis. By matching or stratifying sampling of participants, effects of confounding factors can be adjusted for. When dealing adjustment in data analysis, assess statistics used in study. Most will be some form of multivariate regression analysis to account for confounding factors measured. | Yes | - Male respondents reporting recreational drug use (aOR 1.37, 95% CI 1.01-1.87), drinking alcohol on average three or more days a week (aOR 1.62, 95% CI 1.14-2.28) and anal sex (aOR 1.89, 95% CI 1.35-2.64) increased odds of new heterosexual partners, as were those reporting previous STI diagnosis (aOR 1.69, 95% CI 0.99-2.86) or oral sex (aOR 3.61 95% CI 1.64-3.31), while married or cohabiting men and men registered with GP less likely (aOR 0.35, 95% CI 0.25-0.47, and aOR 0.69, 95% CI 0.51-0.93 respectively). CEE women more likely to have had new sexual partner(s) in past year if reported same sex partner ever (aOR 2.31, 95% CI 1.26-4.26), anal or oral sex in past year (aOR 1.48, 95% CI 1.01-2.15 and aOR 1.76, 95% CI 1.23-2.52 respectively), drinking alcohol more frequently (aOR 1.68, 95% CI 1.02-2.80) and previous STI diagnosis (aOR 1.75, 95% CI 1.13-2.73); less likely if married or cohabiting (aOR 0.28, 95% CI 0.20 to 0.38), from A8 compared with A2 (aOR 0.60, 95% CI 0.37-0.97), and over 24 years (aOR 0.71, 95% CI 0.51-0.98). Male CEE respondents more likely to report at least two partners in past year (aOR 2.1, 95% CI 1.3-2.1) and in past five years (aOR 1.7, 95% CI 1.6-2.6. CEE men more than three times as likely to have paid for sex with a woman (aOR 3.2; 95% CI 2.5-4.0) and twice as likely to inject non-prescribed drugs (aOR 2.2; 95% CI 1.3-3.9). CEE women less likely to report having had an STI (aOR 0.7, 95% CI 0.6-1.0). Consistent condom use in past four weeks higher in CEE sample (aOR 1.3, 95% CI 1.0-1.8). |
| **Were outcomes measured in valid and reliable way?**   - Read methods section of paper. If for e.g. lung cancer is assessed based on existing definitions or diagnostic criteria, then answer likely to be yes. If lung cancer assessed using observer reported, or self‐reported scales, risk of over‐ or under-reporting increased, and objectivity compromised. Importantly, determine if measurement tools used validated instrument which has significant impact on outcome assessment validity. - Having established objectivity of outcome measurement (e.g. lung cancer) instrument, it’s important to establish how measurement conducted. Were those involved in collecting data trained or educated in use of instrument/s? (e.g. radiographers). If more than one data collector, similar in terms of level of education, clinical or research experience, or level of responsibility in piece of research being appraised? | Yes | - Measuring sexual behaviour generally and risky sexual behaviour specifically. Primary outcome measures were at least one new sexual partner in past year (a measure of high-risk sexual behaviour) and whether respondent had a GP (a measure of health service utilisation). - No information on competence of data collectors. |
| **Was appropriate statistical analysis used?**   - As with any consideration of statistical analysis, should consider whether more appropriate alternate statistical method could have been used. Methods section of cohort studies should be detailed enough for reviewers to identify which analytical techniques used (in particular, regression or stratification) and how specific confounders measured. - For studies utilising regression analysis, useful to identify if study identified which variables included and how related to outcome. If stratification was analytical approach used, were strata of analysis defined by specified variables? Also important to assess appropriateness of analytical strategy in terms of assumptions associated with approach as differing methods of analysis based on differing assumptions about data and how it will respond. | Yes | - Χ^2^ and Student’s t, examined associations between variables. Multivariate analysis of factors associated with one or more new heterosexual partner in previous 12 months used a backwards-stepwise model; all variables with p value <0.1 for crude association retained for incorporation in multivariate model. No alternative statistical analysis discussed. - Logistic regression modelling to obtain adjusted ORs (aORs) to control for any variation in age, gender, marital status and education between two data sources. |

| **Evans (2011) Central and East European migrant men who have sex with men: an exploration of sexual risk in the UK** | | |
| --- | --- | --- |
| **Were inclusion criteria in sample clearly defined?**   - Authors should provide clear inclusion and exclusion criteria developed prior to recruiting study participants. Inclusion/exclusion criteria should be specified with sufficient detail and all necessary information critical to study. | Yes | - Cross-sectional survey and used specific strategy for recruiting CEE MSM from across UK. CEE men who have sex with men. Literate men aged 18+ who self-identified as migrants one of 10 CEE countries. |
| **Were study subjects and setting described in detail?**   - Study sample should be described in sufficient detail so that other researchers can determine if comparable to population of interest to them. Authors should provide clear description of population from which study participants selected or recruited, including demographics, location, and time period. | Yes | - CEE men who have sex with men. Literate men aged 18+ who self-identified as CEE migrants. Recruited from two dating websites for gay men in UK. Link placed on homepage of Gay Romeo. While SALLEE project focused on CEE migrants in London, online invite in Gaydar and Gay Romeo extended to whole of UK. Link to questionnaire in Gaydar appeared from March-April 2009 while link on Gay Romeo from April-May 2009. |
| **Was exposure measured in valid and reliable way?**   - Study should clearly describe method of measurement of exposure. Assessing validity requires that 'gold standard' is available to which measure can be compared. Validity of exposure measurement usually relates to whether current measure is appropriate or whether measure of past exposure needed. - Reliability refers to processes included in an epidemiological study to check repeatability of measurements of exposures. These usually include intra-observer reliability and inter- observer reliability. | Yes | - Compared with men living outside London, London men significantly more likely to report paying for sex in UK (15.4% vs 6.8%, p=0.005), had ever been diagnosed with an STI (34.1% vs 22.5%, p=0.003) and had taken recreational drugs in past year (41.5% vs 25.2%, p<0.001). Also less likely to report assortative sexual mixing (18.6% vs 29.1%, p=0.003). - When possible, questions from previously validated questionnaires used to maximise reliability and validity. |
| **Were objective, standard criteria used for measurement of condition?**   - Useful to determine if patients included in study based on either specified diagnosis or definition. More likely to decrease risk of bias. Characteristics another useful approach to matching groups, and studies that did not use specified diagnostic methods or definitions should provide evidence on matching by key characteristics. | Yes | - CEE migrant men who have sex with men included. Health service use as result of risky sexual behaviour that gives rise to STIs. |
| **Were confounding factors identified?**   - Confounding occurred where estimated intervention exposure effect biased by presence of some difference between comparison groups (apart from exposure investigated/of interest). Typical confounders include baseline characteristics, prognostic factors, or concomitant exposures (e.g. smoking). Confounder is difference between comparison groups and it influences direction of study results. A high quality study at level of cohort design will identify potential confounders and measure them (where possible). Difficult for studies where behavioural, attitudinal or lifestyle factors may impact on results. | Yes | - Employment status, age, completed higher education, from Romania or Bulgaria, length of residence in UK, living in London, recruited through Gay Romeo, HIV status, injected drugs, taken recreational drugs, been paid for sex in UK. All factors in Table 4. |
| **Were strategies to deal with confounding factors stated?**   - Strategies to deal with effects of confounding factors may be dealt within study design or in data analysis. By matching or stratifying sampling of participants, effects of confounding factors can be adjusted for. When dealing adjustment in data analysis, assess statistics used in study. Most will be some form of multivariate regression analysis to account for confounding factors measured. | Yes | - Compared with men living elsewhere, London men more likely to have attended a sexual health clinic in UK (53.2% vs 36.6%, p<0.001), to have obtained condoms from health services in UK in past year (32.6% vs 21.1%, p=0.004) and to have tested for HIV (83.0% vs 68.3%, p<0.001). Longer men had been in UK, less likely to report assortative sexual mixing (33.6-14.9%, p<0.001) and more likely to report recreational drug use in past year (31.4-41.4%, p=0.005). Risk of being diagnosed with an STI increased longer men had been in UK, from 27.6% of men who had been in UK for up to one year to 37.2% of men who had been in UK for at least five years (p=0.035); and men who had been in UK for longer also more likely to report ever paying for sex (12.4-18.1%, p=0.026). London men more likely to report being HIV positive (5.6% vs 3.1%, p<0.001). Unprotected Anal Intercourse (UAI) with casual partner of discordant or unknown HIV status more common in HIV positive men (46.9%) than HIV-negative or untested men (18.8% or 27.7%, respectively, p<0.001). |
| **Were outcomes measured in valid and reliable way?**   - Read methods section of paper. If for e.g. lung cancer is assessed based on existing definitions or diagnostic criteria, then answer likely to be yes. If lung cancer assessed using observer reported, or self‐reported scales, risk of over‐ or under-reporting increased, and objectivity compromised. Importantly, determine if measurement tools used validated instrument which has significant impact on outcome assessment validity. - Having established objectivity of outcome measurement (e.g. lung cancer) instrument, it’s important to establish how measurement conducted. Were those involved in collecting data trained or educated in use of instrument/s? (e.g. radiographers). If more than one data collector, similar in terms of level of education, clinical or research experience, or level of responsibility in piece of research being appraised? | Yes | - Incomplete CEE nationality data in UK increases risk of under-representing phenomenon. - No information about competence of analysts. |
| **Was appropriate statistical analysis used?**   - As with any consideration of statistical analysis, should consider whether more appropriate alternate statistical method could have been used. Methods section of cohort studies should be detailed enough for reviewers to identify which analytical techniques used (in particular, regression or stratification) and how specific confounders measured. - For studies utilising regression analysis, useful to identify if study identified which variables included and how related to outcome. If stratification was analytical approach used, were strata of analysis defined by specified variables? Also important to assess appropriateness of analytical strategy in terms of assumptions associated with approach as differing methods of analysis based on differing assumptions about data and how it will respond. | Yes | - Χ^2^, Student’s t test and Mann Whitney U test to examine associations between place of residence and background characteristics, risk behaviour and health service use. Linear-by-linear associations to assess effect of length of time in UK on risk behaviour and health service use, by grouping respondents’ length of stay into yearly intervals up to five years (e.g., up to 1 year, 1-2 years), then over five years. No alternative statistical analysis discussed. - Logistic regression modelling to obtain OR and adjusted OR (aOR) to examine association between UAI with a casual partner and background characteristics, commercial sex, recreational drug use and HIV status. Analysis using SPSS version 12.0. |

| **Evans (2011) Factors associated with genitourinary medicine clinic attendance and sexually transmitted infection diagnosis among central and east European migrants in London** | | |
| --- | --- | --- |
| **Were inclusion criteria in sample clearly defined?**   - Authors should provide clear inclusion and exclusion criteria developed prior to recruiting study participants. Inclusion/exclusion criteria should be specified with sufficient detail and all necessary information critical to study. | Yes | - Eligible respondents for both samples literate men and women aged 18 years or over who self-identified as migrants from one of 10 CEE countries. Eligible respondents born or spent formative years in a CEE country. Length of time in UK not pre-condition for participation. |
| **Were study subjects and setting described in detail?**   - Study sample should be described in sufficient detail so that other researchers can determine if comparable to population of interest to them. Authors should provide clear description of population from which study participants selected or recruited, including demographics, location, and time period. | Yes | - Survey of sexual behaviour conducted among CEE migrants attending two central London GUM clinics (n=299) and community venues in London (n=2,276). Routinely collected data on all attendances of CEE patients and elsewhere at same two GUM clinics analysed from 1 July 2008 to 31 March 2009. SALLEE community sample recruited from range of venues in London over same period. Nine fieldworkers involved in community sample recruitment native speakers of six CEE languages. For GUM sample, recruitment days and times in clinics varied over data collection period. During recruitment times, men and women born in a CEE country asked to participate. Resulting community sample reflects broad cross-section of CEE migrants in London. |
| **Was exposure measured in valid and reliable way?**   - Study should clearly describe method of measurement of exposure. Assessing validity requires that 'gold standard' is available to which measure can be compared. Validity of exposure measurement usually relates to whether current measure is appropriate or whether measure of past exposure needed. - Reliability refers to processes included in an epidemiological study to check repeatability of measurements of exposures. These usually include intra-observer reliability and inter- observer reliability. | Yes | - Questionnaire concentrated on sexual risk behaviour, including sexual practices, numbers of partnerships, using condoms, paying for sex, sexually transmitted infection (STI) (including HIV) and use of sexual health services. - No discussion of intra-observer or inter-observer reliability. |
| **Were objective, standard criteria used for measurement of condition?**   - Useful to determine if patients included in study based on either specified diagnosis or definition. More likely to decrease risk of bias. Characteristics another useful approach to matching groups, and studies that did not use specified diagnostic methods or definitions should provide evidence on matching by key characteristics. | Yes | - Attending two central London GUM clinics and community venues in London. By definition, to be included, must have GU related condition as GUM attendance a key stipulation. |
| **Were confounding factors identified?**   - Confounding occurred where estimated intervention exposure effect biased by presence of some difference between comparison groups (apart from exposure investigated/of interest). Typical confounders include baseline characteristics, prognostic factors, or concomitant exposures (e.g. smoking). Confounder is difference between comparison groups and it influences direction of study results. A high quality study at level of cohort design will identify potential confounders and measure them (where possible). Difficult for studies where behavioural, attitudinal or lifestyle factors may impact on results. | Yes | - Gender, age, country of birth, homosexual/bisexual associated with having new STI diagnosis among CEE patients attending two GUM clinics in London. |
| **Were strategies to deal with confounding factors stated?**   - Strategies to deal with effects of confounding factors may be dealt within study design or in data analysis. By matching or stratifying sampling of participants, effects of confounding factors can be adjusted for. When dealing adjustment in data analysis, assess statistics used in study. Most will be some form of multivariate regression analysis to account for confounding factors measured. | Yes | - Table 1 shows above confounding factors. - In logistic regression analysis adjusting for background characteristics (age, work status, education, relationship status, time in UK, country of birth), all significant associations between sample and sexual risk behaviours listed in Table 3 remained significant (at p<0.001 among women and p<0.01 among men). |
| **Were outcomes measured in valid and reliable way?**   - Read methods section of paper. If for e.g. lung cancer is assessed based on existing definitions or diagnostic criteria, then answer likely to be yes. If lung cancer assessed using observer reported, or self‐reported scales, risk of over‐ or under-reporting increased, and objectivity compromised. Importantly, determine if measurement tools used validated instrument which has significant impact on outcome assessment validity. - Having established objectivity of outcome measurement (e.g. lung cancer) instrument, it’s important to establish how measurement conducted. Were those involved in collecting data trained or educated in use of instrument/s? (e.g. radiographers). If more than one data collector, similar in terms of level of education, clinical or research experience, or level of responsibility in piece of research being appraised? | Yes | - Χ^2^ and student t test to examine associations between factors. They also used regression modelling to obtain odds ratios and adjusted odds ratios. Analysis of routinely collected GUM clinic data conducted for 1 July 2008 to March 2009. Provides context for analysis of SALLEE cross-sectional survey. 29,770 GUM clinic patients attended two clinics at least once during this time and half were men (48.7%). Study did not record country of origin for 8.9% of GUM clinic patients. Of 27,131 men and women whose country of origin was known, 1,373 (5.1%) born in CEE country – 2.9% of men and 7.0% of women. - No information on reliability of data collectors. |
| **Was appropriate statistical analysis used?**   - As with any consideration of statistical analysis, should consider whether more appropriate alternate statistical method could have been used. Methods section of cohort studies should be detailed enough for reviewers to identify which analytical techniques used (in particular, regression or stratification) and how specific confounders measured. - For studies utilising regression analysis, useful to identify if study identified which variables included and how related to outcome. If stratification was analytical approach used, were strata of analysis defined by specified variables? Also important to assess appropriateness of analytical strategy in terms of assumptions associated with approach as differing methods of analysis based on differing assumptions about data and how it will respond. | Yes | - Χ^2^ and student t test to examine associations between factors. Logistic regression modelling to obtain odds ratios and adjusted odds ratios. No alternative statistical methods discussed. - Table 3 showed background characteristics and sexual risk behaviour among SALLEE respondents by gender and type of sample. Background characteristics broken down into age, employment status, whether they have a degree, married or cohabiting, whether they have lived for more than one year in UK and whether born in A2 country. Behavioural variables included number of partners, heterosexual behaviour, homosexual behaviour, sexual risk behaviour, including STI, sex work and drug use. |

| **Ignaszak-Szczepaniak (2009) Reasons for visiting Polish primary care practices by patients aged 18–44 years: the largest emigrating age group** | | |
| --- | --- | --- |
| **Were inclusion criteria in sample clearly defined?**   - Authors should provide clear inclusion and exclusion criteria developed prior to recruiting study participants. Inclusion/exclusion criteria should be specified with sufficient detail and all necessary information critical to study. | Yes | - Study investigated GP visits among 12,535 patients aged 18–44 years registered with GPs of ‘Medicus’ Practice, living in Chodziez town, Western Poland, and surrounding community. |
| **Were study subjects and setting described in detail?**   - Study sample should be described in sufficient detail so that other researchers can determine if comparable to population of interest to them. Authors should provide clear description of population from which study participants selected or recruited, including demographics, location, and time period. | Yes | - Study investigated GP visits among 12,535 patients aged 18–44 years registered with GPs of ‘Medicus’ Practice, living in Chodziez town, Western Poland, and surrounding community. Patients’ details (e.g. name and surname, identification number, age, gender, place of residence) and diagnostic data electronically collected and saved on computer on an internal, separate database. Data collected from June 2005 to May 2006 and patient’s age defined at beginning of data collection in 2005. 22,769 visits to GP practices between June 2005 and May 2006. Age and gender profile of studied population (aged 18–44 years) similar to total Polish population of same age range; therefore, analysed group representative. |
| **Was exposure measured in valid and reliable way?**   - Study should clearly describe method of measurement of exposure. Assessing validity requires that 'gold standard' is available to which measure can be compared. Validity of exposure measurement usually relates to whether current measure is appropriate or whether measure of past exposure needed. - Reliability refers to processes included in an epidemiological study to check repeatability of measurements of exposures. These usually include intra-observer reliability and inter- observer reliability. | No | - Not examining risk factors that may produce certain outcomes. Instead, looking at reasons for GP attendance. |
| **Were objective, standard criteria used for measurement of condition?**   - Useful to determine if patients included in study based on either specified diagnosis or definition. More likely to decrease risk of bias. Characteristics another useful approach to matching groups, and studies that did not use specified diagnostic methods or definitions should provide evidence on matching by key characteristics. | No | - Patients not included in study on basis of specific condition. Study analysed patient records to establish reasons for their visit to GPs to predict possible reasons why Polish patients living abroad may make appointments with GPs in other countries. |
| **Were confounding factors identified?**   - Confounding occurred where estimated intervention exposure effect biased by presence of some difference between comparison groups (apart from exposure investigated/of interest). Typical confounders include baseline characteristics, prognostic factors, or concomitant exposures (e.g. smoking). Confounder is difference between comparison groups and it influences direction of study results. A high quality study at level of cohort design will identify potential confounders and measure them (where possible). Difficult for studies where behavioural, attitudinal or lifestyle factors may impact on results. | Yes | - Age, gender, place of residence. Data from 22,769 visits to GP practices between June 2005 and May 2006 by Polish patients aged 18–44 years collected electronically. Age categorised into three groups (18–24, 25–34 and 35–44 years). |
| **Were strategies to deal with confounding factors stated?**   - Strategies to deal with effects of confounding factors may be dealt within study design or in data analysis. By matching or stratifying sampling of participants, effects of confounding factors can be adjusted for. When dealing adjustment in data analysis, assess statistics used in study. Most will be some form of multivariate regression analysis to account for confounding factors measured. | Yes | - In general, women more likely to visit GP than men. Highest percentage of consultations for 35- to 44-year-old women, while men of same age needed to see GP less frequently. When comparing men by age group, 25–34 age group has highest attendance. Women aged 25–34 years less likely than other women to visit GP. When comparing patients by gender, men in each subgroup were less frequent visitors than women. |
| **Were outcomes measured in valid and reliable way?**   - Read methods section of paper. If for e.g. lung cancer is assessed based on existing definitions or diagnostic criteria, then answer likely to be yes. If lung cancer assessed using observer reported, or self‐reported scales, risk of over‐ or under-reporting increased, and objectivity compromised. Importantly, determine if measurement tools used validated instrument which has significant impact on outcome assessment validity. - Having established objectivity of outcome measurement (e.g. lung cancer) instrument, it’s important to establish how measurement conducted. Were those involved in collecting data trained or educated in use of instrument/s? (e.g. radiographers). If more than one data collector, similar in terms of level of education, clinical or research experience, or level of responsibility in piece of research being appraised? | Yes | - Collecting data electronically allowed GPs to record current individual health problems according to ICD 10 coding system (International Statistical Classification of Diseases and Related Health Problems), which is only accessible disease classification used obligatorily in Poland, irrespective of level of healthcare service. - Data entry and collection by nine participating GPs, instructed to record health problems being managed at the visits. Nothing about their respective competences. |
| **Was appropriate statistical analysis used?**   - As with any consideration of statistical analysis, should consider whether more appropriate alternate statistical method could have been used. Methods section of cohort studies should be detailed enough for reviewers to identify which analytical techniques used (in particular, regression or stratification) and how specific confounders measured. - For studies utilising regression analysis, useful to identify if study identified which variables included and how related to outcome. If stratification was analytical approach used, were strata of analysis defined by specified variables? Also important to assess appropriateness of analytical strategy in terms of assumptions associated with approach as differing methods of analysis based on differing assumptions about data and how it will respond. | Yes | - For patients making visits, Anova to compare mean number of visits per patient by age group and gender. Underlying assumptions necessary for Anova checked using Kolmogorov–Smirnov test for normality with Lilliefors-corrected P-values and Levene’s test for homogeneity of variance. To determine homogenous groups, Tukey’s significant difference test applied to eight mean values (mean number of visits) for each combination of age group and gender. All tests analysed at 5% significance level. No discussion of alternative statistical analysis. - Further analysis indicated a statistically significant interaction between age groups and gender. Women aged 35–44 years visited their GPs more often than those aged 18–24, while men aged 18–24 years visited their GP more often than those aged 35–44. |

| **Leaman (2006) Use of the emergency department by Polish migrant workers** | | |
| --- | --- | --- |
| **Were inclusion criteria in sample clearly defined?**   - Authors should provide clear inclusion and exclusion criteria developed prior to recruiting study participants. Inclusion/exclusion criteria should be specified with sufficient detail and all necessary information critical to study. | Yes | - Patients of Polish origin attending the ED at the Princess Royal Hospital in Telford, UK for six years, up to and including 2005. |
| **Were study subjects and setting described in detail?**   - Study sample should be described in sufficient detail so that other researchers can determine if comparable to population of interest to them. Authors should provide clear description of population from which study participants selected or recruited, including demographics, location, and time period. | Yes | - A novel computer program that assigns a country of origin according to a patient’s given and family names. Using this software the emergency department’s records of the past 6 years were screened, up to and including 2005. Patients of Polish origin were further subdivided into those who were registered with a general practitioner (GP) and those who were not. Of the unregistered Polish patients who attended in 2005, a convenience sample of 90 were further analysed. In particular, the appropriateness of the attendance was assessed by experienced practitioners in emergency medicine and in general practice, using agreed criteria and the notes were reviewed for any comments relating to communication difficulties. |
| **Was exposure measured in valid and reliable way?**   - Study should clearly describe method of measurement of exposure. Assessing validity requires that 'gold standard' is available to which measure can be compared. Validity of exposure measurement usually relates to whether current measure is appropriate or whether measure of past exposure needed. - Reliability refers to processes included in an epidemiological study to check repeatability of measurements of exposures. These usually include intra-observer reliability and inter- observer reliability. |  |  |
| **Were objective, standard criteria used for measurement of condition?**   - Useful to determine if patients included in study based on either specified diagnosis or definition. More likely to decrease risk of bias. Characteristics another useful approach to matching groups, and studies that did not use specified diagnostic methods or definitions should provide evidence on matching by key characteristics. |  |  |
| **Were confounding factors identified?**   - Confounding occurred where estimated intervention exposure effect biased by presence of some difference between comparison groups (apart from exposure investigated/of interest). Typical confounders include baseline characteristics, prognostic factors, or concomitant exposures (e.g. smoking). Confounder is difference between comparison groups and it influences direction of study results. A high quality study at level of cohort design will identify potential confounders and measure them (where possible). Difficult for studies where behavioural, attitudinal or lifestyle factors may impact on results. |  |  |
| **Were strategies to deal with confounding factors stated?**   - Strategies to deal with effects of confounding factors may be dealt within study design or in data analysis. By matching or stratifying sampling of participants, effects of confounding factors can be adjusted for. When dealing adjustment in data analysis, assess statistics used in study. Most will be some form of multivariate regression analysis to account for confounding factors measured. |  |  |
| **Were outcomes measured in valid and reliable way?**   - Read methods section of paper. If for e.g. lung cancer is assessed based on existing definitions or diagnostic criteria, then answer likely to be yes. If lung cancer assessed using observer reported, or self‐reported scales, risk of over‐ or under-reporting increased, and objectivity compromised. Importantly, determine if measurement tools used validated instrument which has significant impact on outcome assessment validity. - Having established objectivity of outcome measurement (e.g. lung cancer) instrument, it’s important to establish how measurement conducted. Were those involved in collecting data trained or educated in use of instrument/s? (e.g. radiographers). If more than one data collector, similar in terms of level of education, clinical or research experience, or level of responsibility in piece of research being appraised? |  |  |
| **Was appropriate statistical analysis used?**   - As with any consideration of statistical analysis, should consider whether more appropriate alternate statistical method could have been used. Methods section of cohort studies should be detailed enough for reviewers to identify which analytical techniques used (in particular, regression or stratification) and how specific confounders measured. - For studies utilising regression analysis, useful to identify if study identified which variables included and how related to outcome. If stratification was analytical approach used, were strata of analysis defined by specified variables? Also important to assess appropriateness of analytical strategy in terms of assumptions associated with approach as differing methods of analysis based on differing assumptions about data and how it will respond. |  |  |
