# Supplementary material for: Understanding how Eastern European migrants use and experience UK health services: a systematic scoping review
Source: BMC Health Serv Res. 2020 Mar 6;20:173. doi: 10.1186/s12913-020-4987-z (PMC7059702; doi:10.1186/s12913-020-4987-z)
Supplement: Supplementary file 5 — Additional file 5: Table S5. Mixed Methods Appraisal Tool (MMAT). [file 12913_2020_4987_MOESM5_ESM.docx]

**Table S5 Mixed Methods Appraisal Tool (MMAT)**

| **Goodwin (2013) Perceived changes in health and interactions with 'the paracetamol force': A multimethod study** | | |
| --- | --- | --- |
| **1. Qualitative** | | |
| 1.1. Are the sources of qualitative data (archives, documents, informants, observations) relevant to address the research question (objective)? | Yes | - Interview data explored Polish migrant experiences of the health service, and the manner in which the experience of the Polish service might influence these experiences and perceptions. - The authors took a pragmatic approach to combining quantitative and qualitative data. It adopts a sequential explanatory mixed methods design, which collects quantitative data first before supplementing it with qualitative data. Quantitative data from the questionnaires informed the design of the semi-structured interviews. Qualitative data explored in detail about the health experiences of Polish migrants in the UK. |
| 1.2. Is the process for analysing qualitative data relevant to address the research question (objective)? | Yes | - The interview data explored Polish migrant experiences of the health service, and the manner in which the experience of the Polish service might influence these experiences and perceptions. - As a supplement to this quantitative analysis, we also questioned those Time 3 participants who had used general practitioners, dentists, or hospital services in the NHS about their satisfaction levels with their treatment. We also asked all Time 3 respondents whether they would prefer to travel to Poland for medical treatment, if monetary concerns were not a restriction. - The second (qualitative) phase of the study builds on these final questions to consider how perceptions about the role of health professionals in Poland influence expectations about treatment by Polish migrants, and their preferences for use of their own health system. Qualitative data provides richer detail about the health experiences of Polish migrants in the UK, helping to better understand the complexity of emotions and motivations involved in seeking medical assistance, and the cultural influences on health-related behaviour (Castro, Kellison, Boyd, & Kopak, 2010; Johnson et al., 2007). |
| 1.3. Is appropriate consideration given to how findings relate to the context, e.g., the setting, in which the data were collected? | Yes | - Study tested a model of the pathways to perceptions of mental and physical health among Polish migrants to the UK. It examines how the experiences of Polish migrants in their home country with their health system influence their use of, and satisfaction with, the UK NHS. - The study adopts a quant-qual mixed method approach *(*Hanson, Cresswell, Clark, Petska, & Creswell, 2005; Johnson et al., 2007) with a structured questionnaire followed by a semi structured interview (e.g., Jakob, 2001). - Study appropriate because questionnaire establishes trends, while semi-structured interviews can explore themes in greater depth. Design enables a dialogue between different research methods (Brannen & Moss, 2012). Data collected from one wave of the quantitative study informs the qualitative study. Data from the qualitative study informs subsequent questionnaire items (Onwuegbuzie & Johnson, 2006). |
| 1.4. Is appropriate consideration given to how findings relate to researchers’ influence, e.g., through their interactions with participants? | Yes | - Relationship between the methods but no real discussion about relationship between researcher and participants. - Quantitative data collected over three waves, with interview data collected over two. Some reciprocation in the questions asked, which enables better integration of data. Consequently, there was an element of sequential triangulation, with one stage influencing the next (Johnson, et al., 2007). Design enables a dialogue between different research methods (Brannen & Moss, 2012). Data collected from one wave of the quantitative study informs the qualitative study. Data from qualitative study informs subsequent questionnaire items (Onwuegbuzie & Johnson, 2006). |
| **2. Quantitative randomised controlled (trials)** | | |
| 2.1. Is there a clear description of the randomisation (or an appropriate sequence generation)? |  |  |
| 2.2. Is there a clear description of the allocation concealment (or blinding when applicable)? |  |  |
| 2.3. Are there complete outcome data (80% or above)? |  |  |
| 2.4. Is there low withdrawal/drop-out (below 20%)? |  |  |
| **3. Quantitative non-randomised** | | |
| 3.1. Are participants (organisations) recruited in a way that minimises selection bias? |  |  |
| 3.2. Are measurements appropriate (clear origin, or validity known, or standard instrument; and absence of contamination between groups when appropriate) regarding the exposure/intervention and outcomes? |  |  |
| 3.3. In the groups being compared (exposed vs. non-exposed; with intervention vs. without; cases vs. controls), are the participants comparable, or do researchers take into account (control for) the difference between these groups? |  |  |
| 3.4. Are there complete outcome data (80% or above), and, when applicable, an acceptable response rate (60% or above), or an acceptable follow-up rate for cohort studies (depending on the duration of follow-up)? |  |  |
| **4. Quantitative descriptive** | | |
| 4.1. Is the sampling strategy relevant to address the quantitative research question (quantitative aspect of the mixed methods question)? | Yes | - Used a variety of means, including banner advertisements on internet portals, and widely distributed leaflets in the places where Polish people frequent, including Polish shops, churches and transport hubs. |
| 4.2. Is the sample representative of the population understudy? | Yes | - Sample drawn from a wide range of locations across the UK. This represented the unusually widespread nature of recent Polish migration to the UK (Burrell, 2009). |
| 4.3. Are measurements appropriate (clear origin, or validity known, or standard instrument)? | Yes | - Some directional assumptions about variables measured in same or subsequent data wave. Expect education and age at arrival to predict language fluency in English at Time 1, while language fluency at Time 1 will predict attitudes to British culture (Pro-British Time 1) and support from British people (closeness to British Time 1 will predict attitudes to British culture (Pro-British Time 1) will in turn predict perceived discrimination (Discrimination Time 2). Attitudes to British culture (Pro-British Time 1) will, in turn, predict perceived discrimination (Discrimination Time 2). Support from Polish and British networks at Time 2 and discrimination at Time 2 and discrimination at Time 2, anticipate predicting SES at Time 3. Latter variable expected to predict perceived changes in physical and psychological health at Time 3. Also expect social support from Polish networks at Time 2 will predict perceived changes in both physical and mental health at Time 3, and support from British networks at Time 2 will predict perceived changes in mental health at Time 3. Expect a direct effect of discrimination Time 2 on mental and physical health at Time 3. Control for the effect of gender and age on perceived changes in physical health. |
| 4.4. Is there an acceptable response rate (60% or above)? | Yes | - The authors suggest that the rate of attrition, which is effectively a response rate for longitudinal data, of over half was acceptable in the context of other longitudinal studies they have cited. - 418 participants at T1, 228 at T2, and 214 at T3. Quantitative data on 172 participants who took part in T1-T3. Half of participants were retained across T1-T3. Dropout rate not particularly large: Markovizky and Samid (2008), for example, in a longitudinal study of new Eastern European migrants to Israel reported 65% attrition rate, while Schwarzer, Hahn, and Schröder (1994), in a study of East German migrants, reported 77% attrition rate over a similar 2-year period. |
| **5. Mixed methods** | | |
| 5.1. Is the mixed methods research design relevant to address the qualitative and quantitative research questions (or objectives), or the qualitative and quantitative aspects of the mixed methods question (or objective)? | Yes | - Study tested a model of the pathways to perceptions of mental and physical health among Polish migrants to the UK. |
| 5.2. Is the integration of qualitative and quantitative data (or results*) relevant to address the research question (objective)? | Yes | - *Quantitative data looked at use of social networks as well as satisfaction with hospitals, GPs and dentists.* Quantitative and qualitative methods complementary. Quantitative survey demonstrated that most participants, given no financial barriers, would prefer to return to Poland for medical treatment. However, interviews explored the factors underpinning this choice. These included the urgency of the situation, the existence (or absence) of a language barrier, the availability of health insurance at work in the United Kingdom, the repercussions for the worker if they had to travel back to Poland, and the availability of support networks in the United Kingdom. |
| 5.3. Is appropriate consideration given to the limitations associated with this integration, e.g., the divergence of qualitative and quantitative data (or results*) in a triangulation design? | Yes | - Limitations reported, but mainly pertaining to quantitative survey response rate, rather than potential divergence of quantitative and qualitative data. - Quantitative data relied on online self-reports. Such reports risk common error variance, which inflates correlations (McBroom & Reed, 1992), although this is usually tempered by longitudinal analyses *(*Podsakoff, MacKenzie, Lee, & Podsakof & Podsakoff, 2003)*.* Overburdening respondents with long questionnaires increases risk of non-response (Sahlqvist et al., 2011). |
| *Criteria for the qualitative component (1.1 to 1.4), and appropriate criteria for the quantitative component (2.1 to 2.4, or 3.1 to 3.4, or 4.1 to 4.4), must be also applied.* | | |
